# NExON-Bayes: a Bayesian approach to network estimation informed by ordinal covariates

**DOI:** 10.1093/bioinformatics/btag345

**Published:** 2026-06-02

**Authors:** Joseph Feest, Hélène Ruffieux, Camilla Lingjærde, Xiaoyue Xi

**Affiliations:** MRC Biostatistics Unit, University of Cambridge, Cambridge CB2 0SR, United Kingdom; MRC Biostatistics Unit, University of Cambridge, Cambridge CB2 0SR, United Kingdom; Department of Mathematics, University of Oslo, Oslo N-0316, Norway; MRC Biostatistics Unit, University of Cambridge, Cambridge CB2 0SR, United Kingdom

## Abstract

**Motivation:**

In heterogeneous disease settings, accounting for intrinsic sample variability is crucial for obtaining reliable and interpretable omic network estimates. However, most graphical model analyses of biomedical data assume homogeneous conditional dependence structures, potentially leading to misleading conclusions. To address this, we propose a joint Gaussian graphical model that leverages sample-level ordinal covariates (e.g. disease stage) to account for heterogeneity and improve the estimation of partial correlation structures.

**Results:**

Our modelling framework, called NExON-Bayes, extends the graphical spike-and-slab framework to account for ordinal covariates, jointly estimating their relevance to the graph structure and leveraging them to improve the accuracy of network estimation. To scale to high-dimensional omic settings, we develop an efficient variational inference algorithm tailored to our model. Through simulations, we demonstrate that our method outperforms the vanilla graphical spike-and-slab (with no covariate information), as well as other state-of-the-art network approaches which exploit covariate information. Applying our method to reverse phase protein array data from patients diagnosed with stage I, II or III breast carcinoma, we estimate the behaviour of proteomic networks as cancer progresses. Our model provides insights not only through inspection of the estimated proteomic networks, but also of the estimated ordinal covariate dependencies of key groups of proteins within those networks, offering a comprehensive understanding of how biological pathways shift across disease stages.

**Availability and implementation:**

A user-friendly R package for NExON-Bayes with tutorials is available on Github at github.com/jf687/NExON, and archived at https://doi.org/10.5281/zenodo.20312938. The source of the dataset used is cited in the relevant section.

## 1 Introduction

Graphical models are useful tools to uncover complex dependence structures between molecular variables and shed light on the biological mechanisms underlying disease. In Gaussian graphical models (GGMs), typically used for the analysis of omic data, the partial correlations between nodes can be derived from the off-diagonal elements of the inverse covariance (precision) matrix. Various approaches for GGM estimation have been developed in the frequentist and Bayesian settings. Frequentist approaches are based on penalized maximum likelihood estimation and include neighbourhood selection (Meinshausen and Bühlmann 2006), the graphical lasso (Friedman *et al*. 2008), and the graphical SCAD (Fan *et al*. 2009). Bayesian methods place a shrinkage prior on the precision matrix entries and include the Bayesian graphical lasso (Wang 2012), the graphical horseshoe (Li *et al*. 2019), and the graphical spike-and-slab (Wang 2015). These methods all assume homogeneous dependence structures across all samples, an assumption that is often unrealistic in practical settings where, e.g. samples might be collected from patients across different conditions, such as different disease severities or treatment regimes.

More recently, methods have been developed to jointly estimate graphical models across multiple networks, reflecting such conditions. Joint methods estimate network structures through sharing information between networks when appropriate, while ideally retaining network-specific differences. Several approaches for joint estimation of multiple GGMs have been proposed. Frequentist approaches extend the penalized likelihood framework to add penalty parameters that encourage similarity between network-specific precision matrices (Guo *et al*. 2011, Ma and Michailidis 2016, Lingjærde and Richardson 2023), whilst Bayesian approaches use priors to encourage the potential sharing of network structures (Peterson *et al*. 2015, Li *et al*. 2019, Lingjærde *et al*. 2024). Such methods have been used, e.g. to estimate the proteomic networks of breast cancer patients with different subtype diagnoses, where the expected similarities between networks are leveraged in the joint estimation (Lingjærde and Richardson 2023).

Existing joint estimation approaches often treat networks as exchangeable, which in some cases can inhibit statistical power. For example, when the covariates used to define subgroups have a natural ordering, the assumption of exchangeability both omits useful information that could be leveraged in the estimation and limits the potential insight into how those covariates affect graphical structure. A related set of more recent approaches build on this idea by making GGMs dependent on covariates. Despite being applicable in a plethora of settings, these approaches do not ensure the positive definiteness of the precision matrix and often lack scalability, which seems to hamper widespread adoption (Ni *et al*. 2022, Niu *et al*. 2024).

Here, we present NExON-Bayes, a Bayesian approach to network estimation informed by ordinal covariates. NexON-Bayes is thus applicable to settings with explicit ordinal covariates or where an ordinal structure can reasonably assumed. These covariates encode auxiliary, non-omic data, which can represent various types of information, such as tumour progression with a covariate taking values in {1,2,3} corresponding to stage I, II, and III cancers, therapeutic response categories with covariate taking values in {1,2,3,4} corresponding to the response category (Eisenhauer *et al*. 2009), or genotype data with covariate taking values in {0,1,2} corresponding to the number of copies of a specified allele. NexON-Bayes builds on the standard graphical spike-and-slab model of Wang (2015), and introduces a sub-model that characterizes the dependence between edge inclusion probabilities and sample-level ordinal covariate data, rather than modelling the entries of the precision matrix, as done in other covariate-dependent approaches. This modelling framework allows us to develop a deterministic inference algorithm that is more scalable than existing alternatives in the literature.

This paper is organized as follows. Section 2 introduces the terminology and notation used throughout the article, our proposed model, and the variational inference algorithm. Next, Section 3 assesses the performance of our approach by benchmarking the model against several competitors through simulations, and investigates its benefits in a real-life setting where we estimate the proteomic networks of breast carcinoma patients. Finally, Section 4 summarizes our contributions and avenues for extensions of this work.

## 2 Materials and methods

### 2.1 Terminology and notation

A graphical model is a probabilistic framework that represents dependencies among a set of random variables. It is represented by G=(V,E), where V={1,…,P} is the set of nodes (variables) and *E* is the set of undirected edges between them. In a Gaussian graphical model (GGM) the nodes represent random variables from a multivariate Gaussian distribution, whilst the edges represent conditional dependence between the variables that they connect. A GGM assumes that *N* samples of *P* variables are independently and identically distributed (i.i.d.) according to


$$y_{n}∼N_{P}(0,\Omega^{-1}),\;n=1,\ldots ,N,$$

where Ω is the positive definite inverse covariance matrix, known as the *precision matrix*. In this work, we consider settings in which each of the *N* samples is characterized by a sample-level ordinal covariate *a* taking values in a set A. To leverage this information, we assume sample homogeneity only within groups defined by equal covariate value, rather than across all samples. Specifically,


(1) $$y_{n}^{\left(a\right)}∼N_{P}(0,{\left(\Omega^{\left(a\right)}\right)}^{-1}),\;n=1,\ldots ,N_{a},$$

where $\Omega^{\left(a\right)}$ is the precision matrix specific to the $N_{a}$ samples with covariate value a∈A [When referring to the covariate value, we use ‘*a*’, and when referring to networks or data that correspond to a covariate value *a* we use ‘(a)’.]. A covariate-specific dataset can then be denoted as $Y^{\left(a\right)}={\left(y_{1}^{\left(a\right)},\ldots ,y_{N_{a}}^{\left(a\right)}\right)}^{⊺}$, an $N_{a}\times P$ matrix whose rows are the individual sample vectors.

Partial correlations between variables can be calculated from these precision matrices, which measure the conditional dependence between variable pairs. Specifically, partial correlation quantifies the correlation between a pair of variables while conditioning on all other variables in the system, thereby isolating direct effects by accounting for potential confounding variables, mediators or colliders. Mathematically, the partial correlation between nodes *i* and *j* in network (a) is given by


$$\rho_{ij}^{\left(a\right)}=-\frac{\Omega_{ij}^{\left(a\right)}}{\sqrt{\Omega_{ii}^{\left(a\right)}\Omega_{jj}^{\left(a\right)}}},\;i\neq j,$$

where $\Omega_{ij}^{\left(a\right)}$ is the *ij*th entry of $\Omega^{\left(a\right)}$. Thus, if an entry of the precision matrix is non-zero, there is conditional dependence between the corresponding nodes (and vice versa). The edge set $E^{\left(a\right)}$ of the corresponding GGM is then given by $E^{\left(a\right)}=\{(i,j)|\Omega_{ij}^{\left(a\right)}\neq 0\}$. Note that because $\Omega^{\left(a\right)}$ is symmetrical, $(i,j)\in E^{\left(a\right)}\Leftrightarrow (j,i)\in E^{\left(a\right)}$.

### 2.2 Graphical spike-and-slab model

To induce sparsity, our proposed approach, NExON-Bayes, relies on a continuous spike-and-slab prior on the off-diagonal elements of each $\Omega^{\left(a\right)}$, similarly as in Wang (2015). This is achieved by using a mixture of two Gaussian distributions, one with very low variance (the spike) and the other with high variance (the slab). A binary edge inclusion indicator is also introduced within this prior, which is denoted as $\delta_{ij}^{\left(a\right)}$, corresponding to the *ij*th entry of $\Omega^{\left(a\right)}$. Edge inclusion is indicated by $\delta_{ij}^{\left(a\right)}=1$, while edge exclusion is indicated by $\delta_{ij}^{\left(a\right)}=0$. The spike-and-slab prior is thus represented as


$$\Omega_{ij}^{\left(a\right)}|\delta_{ij}^{\left(a\right)}∼\delta_{ij}^{\left(a\right)}N(0,\nu_{1}^{2})+(1-\delta_{ij}^{\left(a\right)})N(0,{\left(\nu_{0}^{2}\right)}^{\left(a\right)}),\;\nu_{0}^{2}\ll \nu_{1}^{2},$$

for 1≤i<j≤P, where ${\left(\nu_{0}^{2}\right)}^{\left(a\right)}$ is the network-specific *spike variance* and $\nu_{1}^{2}$ is the *slab variance*. A symmetric matrix is formed by mirroring this lower triangle across the diagonal. Note that a *mixture-of-Gaussians* spike-and-slab prior is used, following Wang (2015), rather than the alternative option of a point-mass spike. This choice preserves positive definiteness in the precision matrix during iterative inference through conjugate updating (George and McCulloch 1997, see Supplementary Materials 1.1 and 1.1.1, available as supplementary data at *Bioinformatics* online).

### 2.3 Dependence on ordinal covariates

NExON-Bayes introduces dependence on an ordinal covariate via a probit submodel placed on the edge inclusion indicator which creates covariate dependence on an edge’s probability of inclusion:


$$\delta_{ij}^{\left(a\right)}|\zeta_{ij},\beta_{ij}∼\text{Bernoulli}\left\{\Phi (\zeta_{ij}+a\beta_{ij})\right\},$$

where Φ(·) is the standard normal cumulative distribution function and *a* is the ordinal covariate. The parameter $\beta_{ij}$ is an edge-specific regression coefficient quantifying the influence of the covariate on the inclusion of edge (i,j). Specifically, $\beta_{ij}=0$ means no relationship between covariate value and edge inclusion probability, i.e. edge (i,j) has the same probability of being present or absent in *all* networks. A positive value of $\beta_{ij}$ means that edge inclusion is more likely in networks with high covariate values and vice versa. In addition, $\zeta_{ij}$ captures the varying baseline inclusion probability of edges and acts as an offset within the probit submodel. We assign a Gaussian prior distribution to $\beta_{ij}$,


$$\beta_{ij}∼N(0,\sigma^{2}),$$

where its hyperparameter $\sigma^{-2}$ is assigned a Gamma prior,


$$\sigma^{-2}∼\text{Gamma}(\alpha_{\sigma },\beta_{\sigma }),\;\alpha_{\sigma }=\beta_{\sigma }=2.$$

Whilst a full multivariate normal could be maintained for the collection of $\beta_{ij}$ coefficients, this mean-field approximation reduces the computational complexity of the algorithm, aiding scalability to high-dimensional settings by reducing runtime and memory requirements. Moreover, the model’s hierarchical structure implicitly preserves some dependency via the precision matrices (see Fig. 2 and analysis in Section 3).

**Figure 1:**
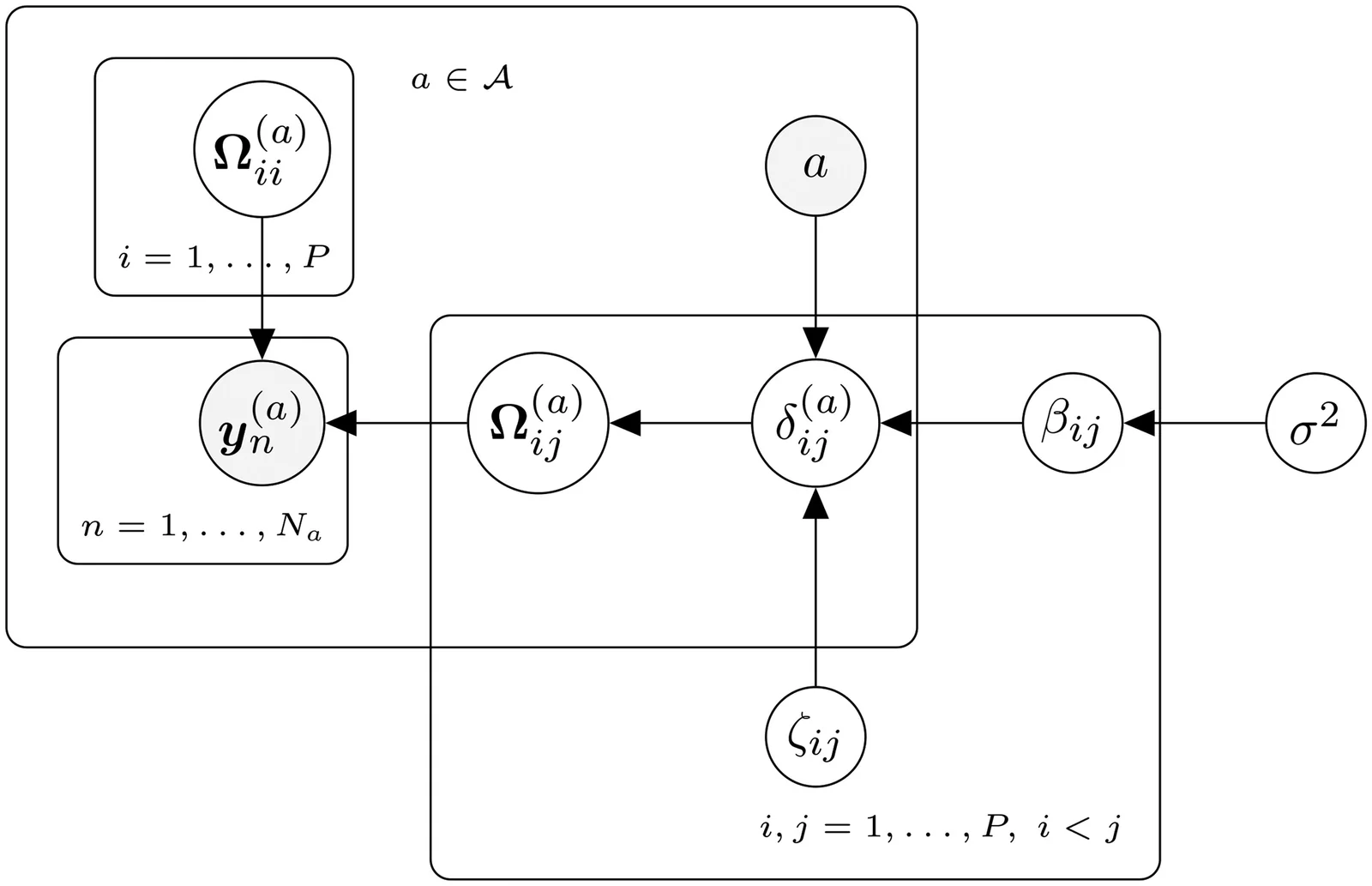
Schematic representation of the NExON-Bayes model. Shaded nodes are observed, and non-shaded are latent variables that are inferred.

Each $\zeta_{ij}$ is also assigned a Gaussian prior,


$$\zeta_{ij}∼N(n_{0},t_{0}^{2}),$$

where the parameters $n_{0}$ and $t_{0}^{2}$ are fixed following Xi and Ruffieux (2025) to prior beliefs about the mean and variance of the number of edges in the networks, and thus the overall sparsity. The hierarchical structure of these priors is presented schematically in Fig. 1.

### 2.4 Variational inference algorithm

Stochastic sampling algorithms like Markov chain Monte Carlo (MCMC) are typically computationally demanding for large graphical models (Wang 2015). Instead, we use deterministic inference algorithms which have been successfully applied in graphical modelling contexts, see, e.g. Li and McCormick (2019), Lingjærde *et al*. (2024), Xi and Ruffieux (2025). Specifically, we develop a variational Bayes expectation conditional maximization (VBECM) algorithm. The true posterior distribution can be written as $p(\underline{\Omega },\Theta |y)$, where $\underline{\Omega }={\left\{\Omega^{\left(a\right)}\right\}}_{a\in A}$ and Θ is the set of all variables outside of the precision matrix. This true distribution is approximated by using a variational distribution with mean-field approximation:


$$q(\underline{\Omega },\Theta )=\prod_{a\in A}q(\Omega^{\left(a\right)})\prod_{a\in A}\prod_{i<j}q(\delta_{ij}^{\left(a\right)},z_{ij}^{\left(a\right)})\prod_{i<j}q(\zeta_{ij})q(\sigma^{-2})\prod_{i<j}q(\beta_{ij}),$$

where

$z_{ij}^{\left(a\right)}$ is introduced by reparametrising $\delta_{ij}^{\left(a\right)}$ as:


$$\begin{array}{c} \delta_{ij}^{\left(a\right)}|z_{ij}^{\left(a\right)}=I\{z_{ij}^{\left(a\right)}>0\},\\ \text{where}\;z_{ij}^{\left(a\right)}|\zeta_{ij},\beta_{ij}∼N(\zeta_{ij}+a\beta_{ij},1). \end{array}$$

The inference problem can then be reframed as finding an approximation to the true distribution, denoted $q(\underline{\Omega },\Theta )$, that is as close as possible to the true posterior distribution by minimizing the reverse Kullback–Leibler divergence. This is equivalent to maximizing the *evidence lower bound* (ELBO),


$$L(q)=E_{q(\underline{\Omega },\Theta )}\{\;\text{log}\;p(Y,\underline{\Omega },\Theta )\}-E_{q(\underline{\Omega },\Theta )}\{\;\text{log}\;q(\underline{\Omega },\Theta )\}$$

(Bishop and Nasrabadi 2006). The optimal distribution for the *n*-th factor of Θ, denoted $q_{n}(\Theta_{n})$ is found by iteratively taking the expectation of the logarithm of the joint distribution over all the other factors,


$$\text{log}\;q_{n}^{*}(\Theta_{n})=E_{q(\underline{\Omega },\Theta \backslash \Theta_{n})}\{\;\text{log}\;p(Y,\underline{\Omega },\Theta )\}+\text{constant},$$

where the constant absorbs all terms not depending on $\Theta_{n}$. Inferring the precision matrices requires more attention, as the variational distribution of each $\Omega^{\left(a\right)}$ is intractable and therefore necessitates a conditional-maximization step where each $\Omega^{\left(a\right)}$ is updated in a blockwise fashion according to Wang (2015). Importantly, this updating scheme ensures that positive definiteness is maintained at each iteration, as detailed in Supplementary Material 1.1.1, available as supplementary data at *Bioinformatics* online. The update rules for each variable, along with their derivations and ELBOs are given in Supplementary Materials 1.1 and 1.2, available as supplementary data at *Bioinformatics* online.

Finally, we use the extended Bayesian Information Criteria (${\text{BIC}}_{\gamma }$) to select the spike variance $\nu_{0}^{2}$. ${\text{BIC}}_{\gamma }$ extends the Bayesian Information Criteria (BIC) with an additional regularization term. The ${\text{BIC}}_{\gamma }$ of a network-specific graphical estimation, $\Omega_{\mathrm{est}.}^{\left(a\right)}$, can be found using the following equation:


$${\text{BIC}}_{\gamma }(\Omega_{\mathrm{est}.}^{\left(a\right)})=\underset{=\text{BIC}}{\underset{︸}{-2\ell (\Omega_{\mathrm{est}.}^{\left(a\right)})+|E^{\left(a\right)}|\;\text{log}(N_{a})}}+4\gamma |E^{\left(a\right)}|\;\text{log}(P),$$

where ℓ(·) is the log likelihood and $\left|E^{\left(a\right)}\right|$ is the number of edges present in estimated network (a) (Foygel and Drton 2010). The regularization parameter γ controls the sparsity of the estimates. Chen and Chen (2008) have shown that, provided that 0≤γ≤1, the asymptotic properties of ${\text{BIC}}_{\gamma }$ are maintained and valid model selection is achieved; we follow the default setting used by these authors, namely γ=0.5, which balances model complexity and selection accuracy in high-dimensional contexts (Foygel and Drton 2010).

To optimize the values of $\nu_{0}$, we use line searches to find a value of $\nu_{0}^{\left(a\right)}$ that minimizes ${\text{BIC}}_{\gamma }(\Omega_{\mathrm{est}.}^{\left(a\right)})$ for each network (a) in the vanilla graphical spike-and-slab model, which gives a strong estimate for expected sparsity.

## 3 Results

### 3.1 Simulated data

We use simulation studies to assess the performance of NExON-Bayes by analysing how well it can recover the true precision matrix from which simulated data is generated. In the main scenario that we consider, linear relations are simulated between probit edge inclusion probabilities and ordinal covariates taking values a∈A={1,2,3,4}. We correspondingly generate four precision matrices, each with P=100 nodes, from which we sample normally distributed data according to Equation (1), where the number of observations for each dataset is $N_{a}=200$. To start, network (1) is simulated as scale-free, with 100 edges (|E|=P) and thus a sparsity of 0.02. ‘Scale-free’ refers to a graph that has a degree distribution that follows a power law, which leads to few nodes having several connected edges (hubs) and most nodes having very few connected edges. This trait emulates generic biological networks, which are often scale-free (Barabási and Bonabeau 2003). The other networks are then created by altering the entries of this first network. Specifically, the zero-valued partial correlations corresponding to 50 edges that are not present in network (1) are simulated to linearly increase with the value of the covariate *a*. Similarly, the non-zero valued partial correlations corresponding to 50 edges that are present in network (1) are simulated to linearly diminish to zero, such that they are not present ($\rho_{ij}=0$) in network (4). The remaining 50 edges present in network (1) are present in all four networks, with unchanged partial correlations. We call edges with increasing partial correlation ‘appearing’ edges and those with decreasing partial correlation ‘disappearing’ edges.

Simulating precision matrices in this way gives rise to an interesting artefact: appearing and disappearing edges have smaller precision matrix entries in networks (2) and (3) and are thus more difficult to uncover. A more detailed description of how these networks are generated can be found in Supplementary Material 2.1, available as supplementary data at *Bioinformatics* online and seen schematically in Fig. 1, available as supplementary data at *Bioinformatics* online.

### 3.2 Borrowing information across networks using joint modelling

We now use this simulated data to compare the performance of NExON-Bayes to the standard single-network Bayesian graphical spike-and-slab model (SSL) (Li and McCormick 2019, Xi and Ruffieux 2025), upon which our extension is built. Specifically, we evaluate whether introducing a direct relationship between a covariate and the posterior probability of edge inclusion (PPI) indeed boosts statistical power—as a first sanity check that the model behaves as it is meant to.

Table 1 indicates improved area under the curve (AUCs) with NExON-Bayes compared to SSL applied to each network individually. This holds for the estimates of all four networks, with a marked difference in the second and third networks—suggesting that the borrowing of information enabled by the joint modelling is particularly beneficial when NExON-Bayes is used for estimating networks with weaker signals.

**Table 1:** AUCs for NExON-Bayes and SSL for each simulated network.^a^

Network	SSL	NExON-Bayes
1	0.966 (0.004)	**0.974** (0.003)
2	0.870 (0.007)	**0.967** (0.003)
3	0.857 (0.009)	**0.946** (0.006)
4	0.980 (0.003)	**0.993** (0.001)

a Standard errors are in brackets, and the highest AUC for each network is shown in bold.

The level of information sharing is controlled by the edge-specific regression coefficient $\beta_{ij}$. Not only is the estimation of $\beta_{ij}$ useful in increasing statistical power, it also provides insight on sample-level heterogeneity in terms of the effect of the sample-level covariate on the graphical structure.

**Figure 2:**
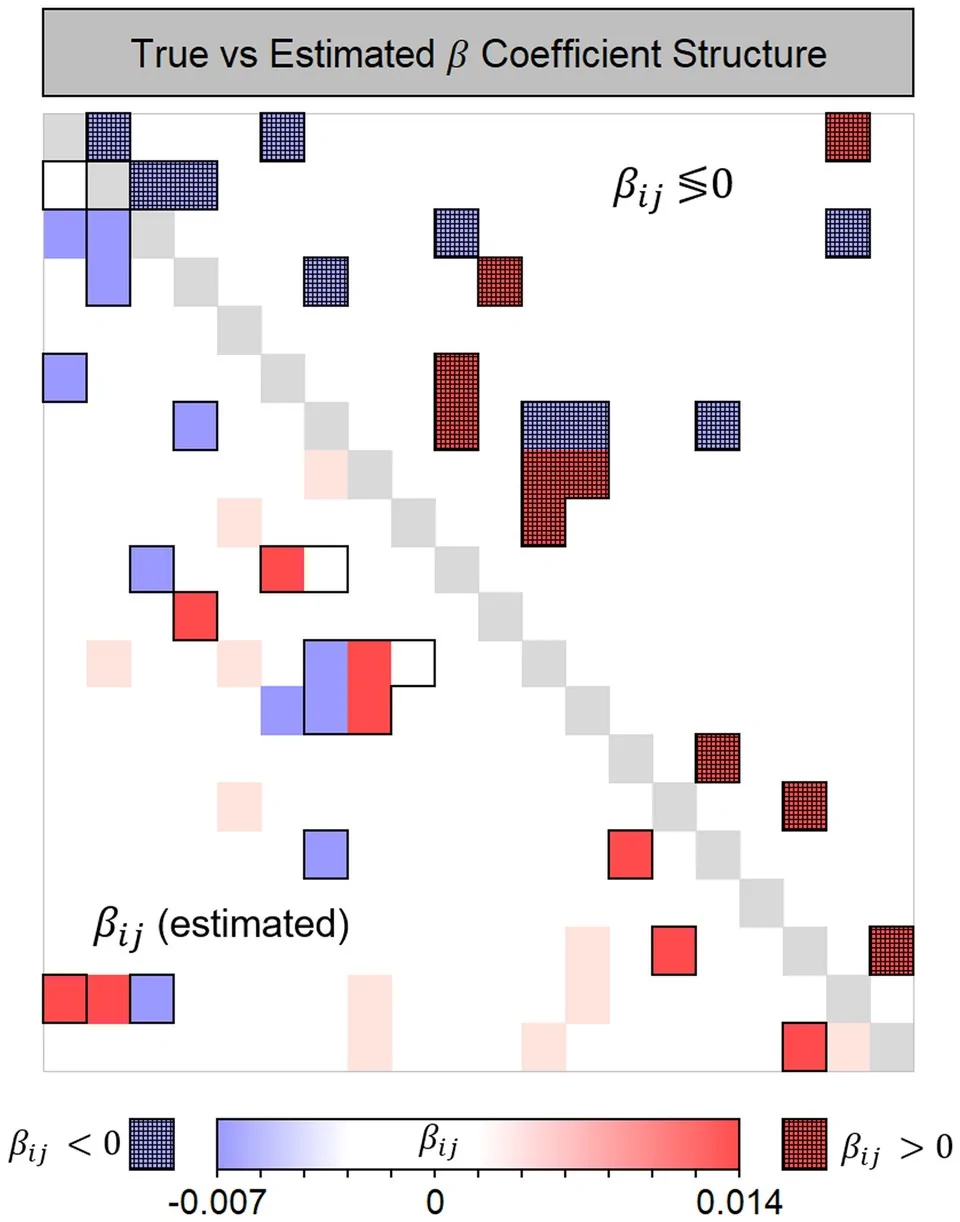
Comparison of estimated and simulated (true) edge-specific regression coefficients in a P=20 simulation setting. The lower diagonal shows the posterior mean estimates of each $\beta_{ij}$ and the upper diagonal shows the true coefficient structure, which, since the data are not generated directly from the model, indicates only the sign of each true $\beta_{ij}$ rather than its exact value. The outline of this true structure is overlain on the estimated structure to ensure a clear comparison.

We now consider a smaller simulated setting (with P=20) focused on assessing the ability of NExON-Bayes to recover edge-specific regression coefficients. Since the simulated data are not generated directly from the model, exact $\beta_{ij}$ values are not analytically identifiable; however, the data generation algorithm imposes known linear correlations between *a* and the precision matrix entries $\Omega_{ij}^{\left(a\right)}$ (see Supplementary Material 2.1, available as supplementary data at *Bioinformatics* online), permitting us to establish the sign of each true $\beta_{ij}$ and test whether this structure is recovered correctly. The results shown in Fig. 2 indicate that, whilst the model does make a mean-field assumption on each $\beta_{ij}$, NExON-Bayes successfully identifies both positive and negative associations with the covariate, information which, as we will illustrate in the cancer data analysis (see *Real-World Application*), can enable a clinically meaningful interpretation of the estimated network structures. Such insights are not captured intrinsically by state-of-the-art single or joint network modelling approaches.

### 3.3 Comparison to existing joint and covariate-dependent approaches

The performance of NExON-Bayes is then compared against three other models that are representative of, or similar to, the methodology incorporated into our proposal. The first is the *covariate dependent graphical estimation* model (covdepGE) of Helwig *et al*. (2024), which models the precision matrix as a function of the covariate. This approach models the dependence on the covariate via the entries of the precision matrix directly, rather than via the PPI of each edge (as is done in NExON-Bayes). The second approach is the *Bayesian Joint Spike-and-Slab Graphical Lasso* (SSJGL) of Li *et al*. (2019), which treats the networks as exchangeable, thereby ignoring any inherent ordering among them. Both of these models are extensions of the graphical spike-and-slab model and use a deterministic inference procedure, as in NExON-Bayes. Lastly, the third is the *multiple graphical horseshoe* (mGHS) of Busatto and Stingo (2025), which treats networks exchangeably and uses a horseshoe prior on precision matrix entries along with an MCMC algorithm.

**Figure 3:**
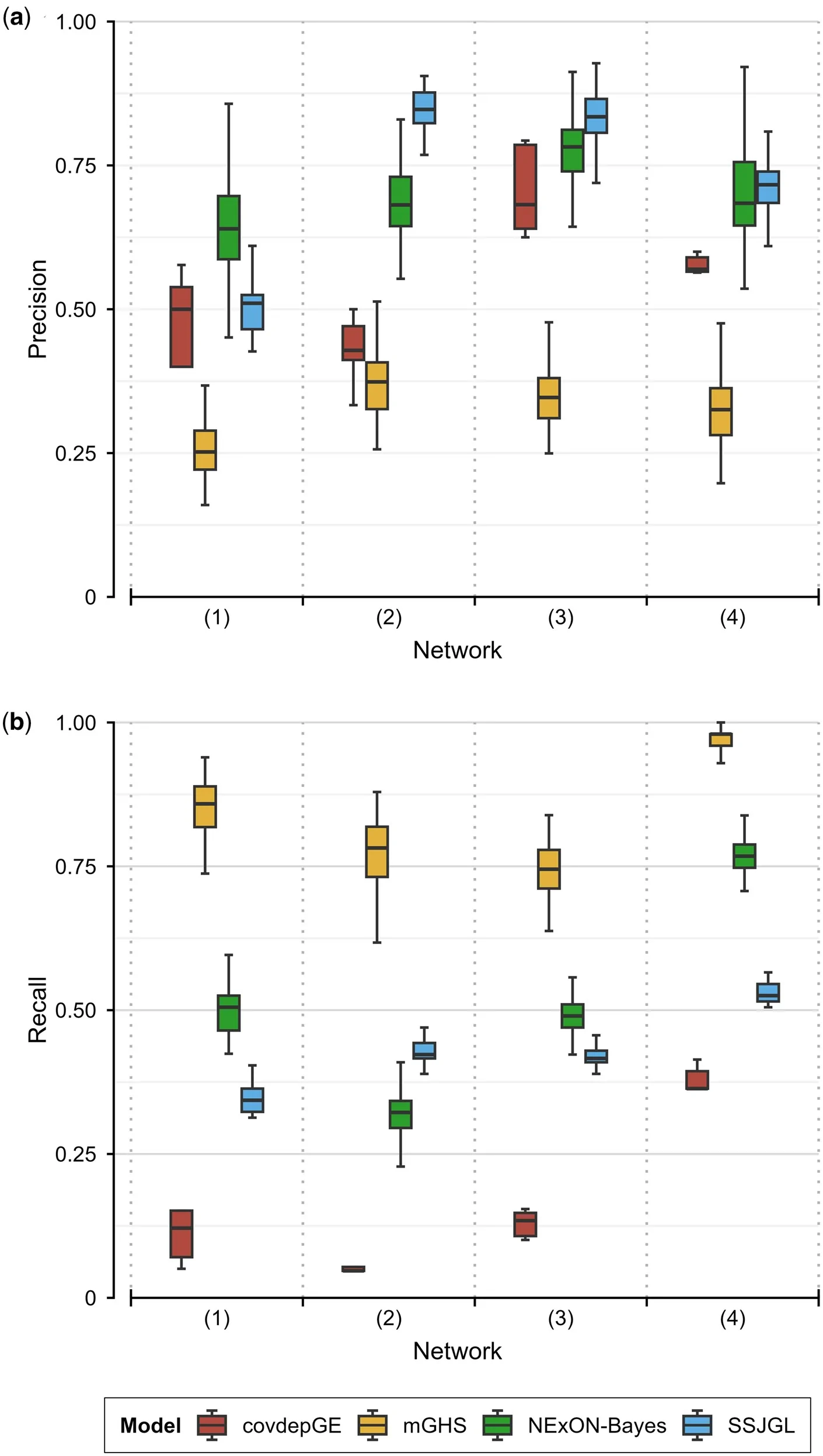
Comparison of performance of covdepGE, mGHS, NExON-Bayes & SSJGL in the simulated scenario outlining (a) precision and (b) recall. The simulated data has P=100 nodes and N=200 samples for each of ∣A∣=4 networks. 50 edges ‘appear’ (absolute value of partial correlation linearly increases with covariate) and 50 edges ‘disappear’ (absolute value of partial correlation linearly decreases with covariate). Performance is assessed over 100 replicates for mGHS, NExON-Bayes and SSJGL and 10 replicates for covdepGE due to excessive runtime for large *N* (>20 h on IntelXeon third generation using 1 CPU (hpc.cam.ac.uk).

We principally assess performance based on *precision*, which measures the proportion of predicted edges that are true positives, and *recall*, which measures the proportion of the true edges that are correctly identified. For NExON-Bayes and covdepGE, we use a 0.5 threshold on the edge PPI to determine edges, based on the median probability model rule (Barbieri and Berger 2004). SSJGL does not estimate PPIs and so edges are determined to be present when the corresponding precision matrix entries are non-zero. For mGHS, we follow the $t^{\alpha }$ thresholding rule introduced by the authors (Busatto and Stingo 2025), which uses a Metropolis-within-Gibbs step to update the threshold parameters. Figure 3 shows the precision and recall of the estimates of each model for each network; for full results that include Matthews correlation coefficient (MCC), accuracy and sparsity, see Supplementary Material 2.3, Table 1, available as supplementary data at *Bioinformatics* online. NExON-Bayes outperforms covdepGE with respect to precision, recall, MCC and accuracy. The poor performance of covdepGE in this setting can be explained by its overly sparse estimates. By only predicting a low number of edges, the total number of true positive edges will remain low, leading to very poor recall. The precision achieved is also the lowest of any model. Furthermore, covdepGE scales particularly poorly with the number of observations *N*, as it models the precision matrix as a continuous function of the covariate, leading to |A|×N=600 precision matrix estimates rather than |A|=4 estimates returned by each of the other models.

SSJGL and NExON-Bayes perform much more similarly in comparison, yet SSJGL estimates networks with slightly higher precision but much lower recall in most cases, particularly in networks (1) and (4). This is because SSJGL infers a shared similarity structure across all networks and uses it to inform the network estimates. In this simulated setting, edges that ‘appear’ are mutual to networks (2), (3), and (4), whilst edges that ‘disappear’ are mutual to networks (1), (2), and (3). Therefore, these edges are often estimated as present in all networks even if they are absent in networks (1) and (4) due to the model’s tendency to over-estimate the level of similarity. This is explored further in Supplementary Material 2.2, available as supplementary data at *Bioinformatics* online.

mGHS exhibits the opposite problem to covdepGE, in that it estimates very dense networks, indicating poor convergence. It achieves the highest recall of any model, which is a misleading result common for such overly dense estimations. The precision of mGHS is the lowest of any model, leading to low accuracy compared to other models. mGHS performs far better in lower dimensional settings (see Supplementary Material 2.5, available as supplementary data at *Bioinformatics* online), suggesting poor scalability.

Overall, this study exemplifies the advantages of NExON-Bayes in practice. To obtain a more complete assessment of NExON-Bayes’ performance and test its robustness, three further simulation studies are explored that, to a varying degree, deviate from the specification of the model. Whilst the performance of NExON-Bayes worsens in misspecified frameworks (as expected), NExON-Bayes is shown to be robust in these settings by maintaining good performance metrics relative to its competitors. The full analysis of these additional simulation studies can be found in Supplementary Material 2.4, available as supplementary data at *Bioinformatics* online.

An analysis of computational runtimes indicates that NExON-Bayes performs inference more quickly than the other joint models that were tested (covdepGE, mGHS, SSJGL) within the range 10≤P≤250. For up to 8 networks with up to 150 variables (P≤150), NExON-Bayes runs in <30 min and in <2 s for P≤50, which is between 1 and 2 orders of magnitude quicker than its competitors. A more complete analysis is given in Supplementary Material 2.5, available as supplementary data at *Bioinformatics* online.

### 3.4 Estimating stage-wise proteomic networks in breast carcinoma

We consider proteomic data from TCGA (The Cancer Genome Atlas 2026; cancer.gov), specifically, reverse phase protein array data of patients that have been diagnosed with stage I, II, and III breast carcinoma [Stage I BRCA refers to tumours that have not spread beyond the breast and are still small in size, stage II refers to slightly larger tumours that may have spread to 1–3 lymph nodes, whilst stage III cancer refers to cancer that has spread to the ‘lymph nodes, the skin of the breast or the chest muscle’ and is larger in size (macmillan.org)] (BRCA). These data have been preprocessed using replicate-based normalization (Akbani *et al*. 2014), a commonly used method for omic data which eliminates unwanted batch effects whilst maintaining approximate Gaussianity, and contain the expression levels of P=131 target proteins for $N_{1}=113$ stage I, $N_{2}=429$ stage II and $N_{3}=140$ stage III individuals. We treat tumour stage as our ordinal covariate capturing patient heterogeneity. Although factors like hormonal status also contribute to heterogeneity, we focus on tumour stage as an ordinal covariate to capture broad trends across disease progression. Our aim is to characterize how tumour severity impacts the proteomic network and whether conditional dependencies between protein pairs strengthen or weaken with tumour progression.

We firstly validate the estimations made by NExON-Bayes by consulting the STRING database (Szklarczyk *et al*. 2023), which contains protein-protein interactions that have been experimentally validated to a given confidence level. We calculate the percentage of pathways that are found by NExON-Bayes and SSL that have been validated to a 90% confidence threshold and show that NExON-Bayes finds a higher percentage of these validated pathways than SSL for all three BRCA stages. Another useful analysis is to identify the ‘hub’ proteins, which we define as proteins whose node degree exceeds the 90th percentile, in the networks estimated by NExON-Bayes. This can shed light on which proteins are prominent in which stages of BRCA. For example, NExON-Bayes identifies *GATA binding protein 3*, *Estrogen Receptor* α (ERα) and *fibronectin* to have degrees in the top 5% in all three stages, whilst *Inositol polyphosphate-4-phosphatase (INPP4B)* is identified as a hub protein in only the stage III: *INPP4B* inactivation is known to be common in triple-negative breast cancer cases (Rodgers *et al*. 2021); see Supplementary Materials 3.1 and 3.2, available as supplementary data at *Bioinformatics* online for full results of both of these analyses.

**Figure 4:**
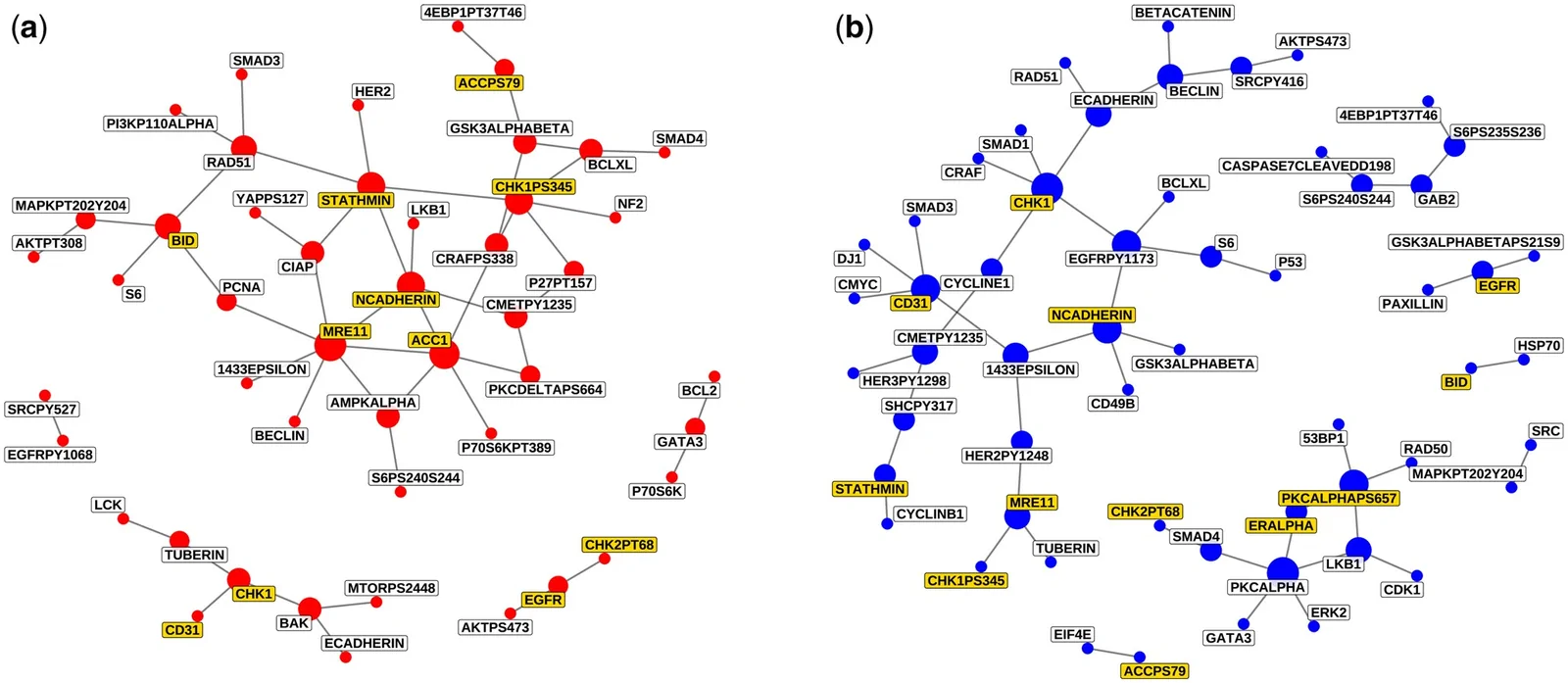
Subnetworks showing (a) the 50 edges with the highest posterior mean $\beta_{ij}$ and (b) the 50 edges with the lowest posterior mean $\beta_{ij}$ obtained by NExON-Bayes when estimating BRCA proteomic networks. Larger node size corresponds to higher node degree. Proteins highlighted are those above the 90th percentile where the *i*th protein is ranked by the sum of the absolute values of all adjacent $\beta_{ij}$s, analogous to a weighted node degree in the full graph.

NExON-Bayes’ framework provides unique insights into molecular networks by estimating the relation between the covariate and the conditional dependence. In essence, inspecting the edges corresponding to the largest absolute posterior means of $\beta_{ij}$ could help identify biological processes that characterize tumour progression when used to estimate the proteomic networks of BRCA patients. Figure 4 shows the subnetworks consisting of the edges corresponding to the 50 largest negative values (in absolute terms) and the 50 largest positive values of the posterior mean estimate of $\beta_{ij}$ estimated by NExON-Bayes.

To further analyse the posterior mean estimates $\beta_{ij}$, we perform a ranked *gene set enrichment analysis* (GSEA), where genes are ranked by the sum of the absolute values of the estimated regression coefficients between the proteins which they encode and all other proteins (with positive and negative networks considered separately as in Fig. 4). The ranked lists are then evaluated to determine whether genes from predefined, biologically curated sets cluster towards the ranking extremes, indicating that membership in those sets is associated with stronger estimated covariate effects (Subramanian *et al*. 2005).

The results of this analysis find several cancer-related pathways to be enriched, centred on apoptosis and cellular stress response. The largest is the *apoptosis modulation and signalling* pathway, which governs programmed cell death—a process cancer cells evade to survive and proliferate (Plati *et al*. 2011). The *TNF*α  *signalling* pathway is also enriched; as a proinflammatory cytokine, TNFα can either promote or inhibit tumour progression depending on context (Valenzuela-Cardenas and Nowicki 2026). Finally, the *ATM Protein signalling* gene set is enriched in the positive network; ATM mutations impair DNA damage response and increase malignancy susceptibility (Varadhan *et al.* 2024).

As a means of comparison, we perform the same analysis using the GraphR model of Chen *et al.*, a graphical model that accounts for covariate information and adopts a decoupling strategy, wherein $\beta_{ij}$ coefficients are estimated separately before applying a variational Bayes inference algorithm. GraphR also enforces sparsity through a spike-and-slab prior on the precision matrices and uses $\beta_{ij}$ to represent the effect of the covariate on edge-specific regression (Chen *et al*. 2025). This is similar to NExON-Bayes and thereby provides a suitable comparison.

The GSEA based on the $\beta_{ij}$ of GraphR identifies several gene sets related to the EGFR family of receptor tyrosine kinases—including *ErbB protein signalling*—which are frequently upregulated in cancers including breast and colorectal cancer (Wee and Wang 2017). ErbB in particular is more prominently expressed in metastatic (stage IV) than primary (stage I) breast cancer (Xue *et al.* 2006). The GSEAs performed for each model find very similar numbers of enriched sets: 23 enriched in the estimations of GraphR, versus 22 in the estimations of NExON-Bayes, where a 0.05 threshold on the nominal *P*-value of each is used, along with a 0.25 false discovery rate threshold. The full results of both GSEAs can be found in Tables 6 and 7, available as supplementary data at *Bioinformatics* online.

The pathways recovered by NExON-Bayes, including apoptosis modulation, TNFα signalling and ATM signalling, reflect core cancer hallmarks that have all been implicated in breast cancer biology and, in several cases, in stage advancement and metastatic potential (Plati *et al*. 2011,  Varadhan *et al.* 2024). These differ in character from the EGFR and ErbB receptor signalling pathways recovered by GraphR, which correspond to more localized, well-characterized stage-associated mechanisms. Rather than viewing these as contradictory, we propose that the two methods may be sensitive to different aspects of the covariate-edge relationship. GraphR, by decoupling estimation, may preferentially recover strong, localized covariate effects on specific edges which here manifests as prominent receptor signalling pathways with known stage associations. NExON-Bayes, by jointly estimating the full network and covariate effects, may instead capture distributed shifts in conditional dependence structure across stages, which present as stress- and damage-response pathways. This effect is likely exacerbated by the differing strength of sparsity constraint of each model: GraphR yields non-zero estimates of $\beta_{ij}$ for 8.6% of edges compared to 18.6% for NExON-Bayes.

The discordance in enriched gene sets between the two models also reflects the sensitivity of GSEA to the precise ordering of proteins across the full ranked list, since small differences in upstream estimates can amplify into divergent enrichment outcomes. To probe this, we applied Fisher’s exact tests comparing the top-ranked proteins identified by NExON-Bayes and GraphR across a range of thresholds and ranking criteria. The tests return consistently significant overlap (Table 5, available as supplementary data at *Bioinformatics* online), indicating that the two models agree on a broadly similar set of highly ranked proteins. This protein-level concordance is consistent with the interpretation that the divergence in GSEA results reflects rank-ordering sensitivity rather than a fundamental disagreement in biological signal.

Whilst in this analysis there is no quantitative criterion to ascertain which strategy (coupling versus decoupling) outperforms the other, these results show that both can find several relevant gene sets. The ability to uncover these gene sets through encapsulating sample-level heterogeneity effectively in a joint model is an important practical advantage of NExON-Bayes (and GraphR). We have shown that, by taking this joint approach, interesting validated or candidate disease mechanisms are uncovered in a disease which progresses through defined stages. These mechanisms warrant dedicated investigation beyond the scope of this paper.

## 4 Discussion

We have introduced a new method, NExON-Bayes, that jointly estimates multiple graphical models while accounting for sample-level heterogeneity as encapsulated in auxiliary ordinal covariate information. We show that, for such auxiliary data, NExON-Bayes’ formulation better captures sample-level heterogeneity than network estimation models that use continuous covariates or treat pre-defined groups of samples in an exchangeable manner, without resorting to covariate information. Moreover, thanks to its efficient deterministic inference procedure, our approach is more scalable and suited to the analysis of several current real data settings. Applying our novel methodology to the TCGA breast cancer dataset reveals new molecular pathways that characterize tumour stage progression, which could lead to new hypotheses about plausible oncogenic mechanisms.

Several avenues for future work could be considered. First, the current prior formulation assumes a linear relationship between the ordinal variable and the probability of edge inclusion—relaxing this linearity assumption through an alternative monotonic parametric formulation may enhance both the model performance and its biological relevance. Secondly, the model could in principle be easily extended to accommodate multiple covariates. We did not pursue this extension here, however, as its practical usefulness is limited: indeed, it would require specifying a separate network for every combination of discrete covariate levels, which would reduce statistical power by fragmenting the sample across many networks. This would also hamper interpretability, as by combining multiple covariates to define networks, they lose their clear ordinality. Next, by using a deterministic inference algorithm, the model does not explicitly represent the full posterior distribution and may therefore underestimate posterior uncertainty (Giordano *et al*. 2018). In principle, stochastic inference algorithms can provide more faithful uncertainty quantification, provided convergence is reached. Accordingly, an interesting direction for future work is to investigate inference procedures that yield richer uncertainty quantification, for instance via hybrid deterministic–stochastic approaches, while maintaining a reasonable trade-off between computational cost and inferential accuracy.

Another interesting extension concerns the treatment of missing data. Whilst NExON-Bayes does not intrinsically deal with missingness, several imputation methods are available for omics data (Lazar *et al*. 2016, Qiu *et al*. 2020). A natural extension to this work would be to instead incorporate model-based Bayesian imputation directly within NExON-Bayes by extending the model hierarchy to explicitly describe the observational process. Lastly, as is typical for joint network estimation models, unequal sample sizes can lead to varying levels of sparsity in the estimated networks, reflecting differences in statistical power. This behaviour can in fact be desirable, as smaller sample sizes naturally yield sparser estimates, reducing the risk of false positives. However, if the goal is to isolate true biological variation independent of sample size effects, this pattern can be adjusted for—though doing so will require methodological advances beyond current approaches (Das *et al*. 2020).

NExON-Bayes offers a principled framework for joint network inference that incorporates sample-level covariates. By uncovering both shared and covariate-dependent patterns of conditional dependence, it enables the systematic characterization of sample-level heterogeneity and supports biologically grounded hypothesis generation for downstream validation.

## Supplementary Material

btag345_Supplementary_Data

## Data Availability

For the purpose of open access, the authors have applied a Creative Commons Attribution (CC BY) license to any Author Accepted Manuscript version arising. Breast carcinoma proteomics data are freely available from https://www.cancer.gov/ccg/research/genome-sequencing/tcga.
