# Supplementary material for: NExON-Bayes: a Bayesian approach to network estimation informed by ordinal covariates
Source: Bioinformatics. 2026 Jun 2;42(9):btag345. doi: 10.1093/bioinformatics/btag345 (PMC13568479; doi:10.1093/bioinformatics/btag345)
Supplement: Supplementary file 1 [file btag345_supplementary_data.pdf]

Supplementary Material

NExON-Bayes: A Bayesian approach to network estimation informed by ordinal covariates

Joseph Feest[1], Hélène Ruffieux[1], Camilla Lingjærde[2], Xiaoyue Xi[1]
[1]MRC Biostatistics Unit, University of Cambridge
[2]Department of Mathematics, University of Oslo

Contents

1 Fast deterministic inference **2**
1.1 Update steps for the VBECM algorithm used by NExON-Bayes . . . 2
1.1.1 Maintaining positive definiteness in the precision matrix . . . 3
1.2 Evidence Lower Bounds (ELBOs) . . . 4

2 Addendum to simulation experiments **5**
2.1 Simulating positive-definite precision matrices with appropriate sample-level heterogeneity 5
2.2 Further analysis of NExON-Bayes' ability to distinguish heteregenous samples . . . 6
2.3 Full tabulated results of the main simulation . . . 7
2.4 Simulations under misspecified settings . . . 7
2.5 Computational runtimes . . . 9
2.5.1 Models restricted by runtime . . . 11

3 Addendum to BRCA application **12**
3.1 Evidence found in STRING database . . . 12
3.2 Hub Proteins . . . 12
3.3 Agreement of top proteins in ranked summary vectors . . . 13
3.4 Enriched gene sets found by NExON-Bayes and GraphR . . . 14

List of Figures

S1 Simulated conditional dependence networks used in the main simulations . . . 5
S2 Comparison of estimated partial correlations for network (2) against network (1) using NExON-Bayes and SSJGL. . . . 6
S3 Computational runtimes for covdepGE, mGHS, NExON-Bayes, SSJGL, and SSL across increasing numbers of networks and a range of dimensions . . . 10
S4 Computational runtimes for GGMx and PxG in settings with 4 networks and a range of dimensions . . . 11

List of Tables

S1 Performance metrics for the main simulation . . . 8
S2 Results of the additional simulations . . . 9
S3 Percentage of edges estimated by SSL and NExON-Bayes in the STRING dataset. . . . 12
S4 Hub proteins found by NExON-Bayes within the BRCA proteomic data . . . 12
S5 Results of Fisher's exact tests on prominent proteins in NExON-Bayes and GraphR estimates 13
S6 Gene sets found to be enriched in the β_{NExON} network estimations made by NExON-Bayes 14
S7 Gene sets found to be enriched in the $\boldsymbol{\beta}_{\text{GraphR}}$ network estimations made by GraphR . . . 15

1 Fast deterministic inference

1.1 Update steps for the VBECM algorithm used by NExON-Bayes

In this section, we present the update steps for the variational Bayes expectation conditional maximisation (VBECM) algorithm used by NExON-Bayes that is adapted from Xi and Ruffieux [2024]. The full model is given as follows. For each ordinal covariate $a \in \mathcal{A}$, let $\boldsymbol{Y}^{(a)} = (\boldsymbol{y}_1^{(a)}, ..., \boldsymbol{y}_{N_a}^{(a)})$ denote the observations across P nodes in a graph. The model then assumes the following priors:

$$\begin{aligned}
\boldsymbol{y_n}^{(a)} &\overset{\text{i.i.d.}}{\sim} \mathcal{N}_P(\boldsymbol{0}, (\boldsymbol{\Omega}^{(a)})^{-1}), \quad \boldsymbol{\Omega}^{(a)} \in \mathcal{M}^+, \quad n = 1, \dots, N_a, \quad a \in \mathcal{A}, \\
\boldsymbol{\Omega}_{ii}^{(a)} &\sim \text{Exp}\,(\lambda/2), \quad i = 1, \dots, P, \\
\boldsymbol{\Omega}_{ij}^{(a)} \mid \delta_{ij}^{(a)} &\sim \delta_{ij}^{(a)} \mathcal{N}(0, \nu_1^2) + (1 - \delta_{ij}^{(a)}) \mathcal{N}(0, \nu_0^2), \quad \nu_0 \ll \nu_1, \quad 1 \le i < j \le P, \\
\delta_{ij}^{(a)} \mid a, \zeta_{ij}, \beta_{ij} &\sim \text{Bernoulli}(\Phi(\zeta_{ij} + a\beta_{ij})), \\
\zeta_{ij} &\sim \mathcal{N}(n_0, t_0^2), \quad \beta_{ij} \sim \mathcal{N}(0, \sigma^2) \quad \text{and} \quad \sigma^{-2} \sim \text{Gamma}(\alpha_\sigma, \beta_\sigma),
\end{aligned}$$

where $\mathcal{M}^+$ is the set of symmetric positive definite matrices, $\lambda, \nu_0, \nu_1, n_0, t_0^2$ are all fixed variables, $\Phi(.)$ is the cumulative standard normal distribution function and initially $\alpha_\sigma = \beta_\sigma = 2$.

To develop a variational inference algorithm for the model, we first approximate the posterior distribution to ensure that it is tractable. To do this, independence assumptions between variables are made where possible. In full:

$$\begin{aligned}
&q(\{\boldsymbol{\Omega}^{(a)}\}_{a\in\mathcal{A}}, \{\boldsymbol{\delta}^{(a)}\}_{a\in\mathcal{A}}, \{\boldsymbol{z}^{(a)}\}_{a\in\mathcal{A}}, \{\zeta_{ij}\}_{i<j}, \{\beta_{ij}\}_{i<j}, \sigma^{-2}) \\
&\qquad = \prod_{a\in\mathcal{A}} q(\boldsymbol{\Omega}^{(a)}) \prod_{a\in\mathcal{A}} \prod_{i<j} q(\delta_{ij}^{(a)}, z_{ij}^{(a)}) \prod_{i<j} q(\zeta_{ij}) q(\sigma^{-2}) \prod_{i<j} q(\beta_{ij}).
\end{aligned}$$

The variational distribution $q(\delta_{ij}^{(a)}, z_{ij}^{(a)})$ follows

$$q(\delta_{ij}^{(a)}, z_{ij}^{(a)}) \quad = \quad q(z_{ij}^{(a)} \mid \delta_{ij}^{(a)}) q(\delta_{ij}^{(a)}), \quad a \in \mathcal{A}, \quad 1 \le i < j \le P,$$

with

$$z_{ij}^{(a)} \mid \delta_{ij}^{(a)}, \boldsymbol{Y} \sim \quad \mathcal{TN}\left\{(\alpha_{ij}^{(a)})^{(1)}, 1, (-1)^{1-\delta_{ij}^{(a)}} z_{ij}^{(a)} > 0\right\}, \quad \delta_{ij}^{(a)} \mid \boldsymbol{Y} \sim \text{Bernoulli}\left((\delta_{ij}^{(a)})^{(1)}\right),$$

where $\mathcal{TN}$ denotes a truncated normal distribution, the superscripts $^{(1)}$ and $^{(2)}$ denote the first, respectively second moments of the variable they are applied to,

$$\begin{aligned}
(\alpha_{ij}^{(a)})^{(1)} =& (\zeta_{ij})^{(1)} + a(\beta_{ij})^{(1)}, \\
\frac{1}{(\delta_{ij}^{(a)})^{(1)}} =& 1 + \exp\left[\log\left(\frac{\nu_1}{\nu_0}\right) + \frac{(\boldsymbol{\Omega}_{ij}^{(a)})^2}{2}\left(\frac{1}{\nu_1^2} - \frac{1}{\nu_0^2}\right) + \log\left\{1 - \Phi\left((\alpha_{ij}^{(a)})^{(1)}\right)\right\} - \log\Phi\left((\alpha_{ij}^{(a)})^{(1)}\right)\right].
\end{aligned}$$

The first two moments of $z_{ij}^{(a)}$ are

$$\begin{aligned}
(z_{ij}^{(a)})^{(1)} =& (\alpha_{ij}^{(a)})^{(1)} + M\left((\alpha_{ij}^{(a)})^{(1)}, 0\right) + (\delta_{ij}^{(a)})^{(1)}\left\{M\left((\alpha_{ij}^{(a)})^{(1)}, 1\right) - M\left((\alpha_{ij}^{(a)})^{(1)}, 0\right)\right\}, \\
(z_{ij}^{(a)})^{(2)} =& (\alpha_{ij}^{(a)})^{(1)} (z_{ij}^{(a)})^{(1)} + 1,
\end{aligned}$$

where $M(\mu, \gamma)$ is the inverse Mills ratio,

$$M(\mu, \gamma) = (-1)^{1-\gamma} \frac{\varphi(\mu)}{\Phi(\mu)^\gamma \left\{1 - \Phi(\mu)\right\}^{1-\gamma}}, \quad \mu \in \mathbb{R}, \quad r = 0, 1,$$

$\varphi(\cdot)$ is the standard normal density and $\Phi(\cdot)$ is the standard normal cumulative distribution function. The variational distribution of ζ_{ij} is

$$\zeta_{ij} \sim \mathcal{N}(\mu_{\zeta_{ij}}, \sigma^2_{\zeta_{ij}}),$$

where

$$\begin{aligned}
\mu_{\zeta_{ij}} &= \sigma^2_{\zeta_{ij}} \left(\sum_{a\in\mathcal{A}} (z_{ij}^{(a)})^{(1)} - (\beta_{ij})^{(1)} \sum_{a\in\mathcal{A}} a + \frac{n_0}{t_0^2} \right), \\
\sigma^{-2}_{\zeta_{ij}} &= T + \frac{1}{t_0^2}.
\end{aligned}$$

In addition, we find

$$\beta_{ij} \mid \boldsymbol{Y} \sim \mathcal{N}(\mu_{\beta,ij}, \sigma^2_{\beta,ij}),$$

where

$$\begin{aligned}
\mu_{\beta,ij} &= \sigma^2_{\beta,ij} \left(\sum_{a\in\mathcal{A}} a(z_{ij}^{(a)})^{(1)} - (\zeta_{ij})^{(1)} \sum_{a\in\mathcal{A}} a \right), \\
\sigma^{-2}_{\beta,ij} &= \sum_{a\in\mathcal{A}} a^2 + \left(\sigma^{-2}\right)^{(1)}.
\end{aligned}$$

The optimal variational distribution of σ^{-2} is

$$\sigma^{-2} \mid \boldsymbol{Y} \sim \text{Gamma}(\alpha_\sigma, \beta_\sigma),$$

where

$$\begin{aligned}
\alpha_\sigma &= \frac{P(P-1)}{4} + a_\sigma, \quad \beta_\sigma = \frac{1}{2}\sum_{i<j} \sigma^2_{\beta_{ij}} + b_\sigma, \\
\left(\sigma^{-2}\right)^{(1)} &= \alpha_\sigma/\beta_\sigma, \quad \left\{ \log\left(\sigma^{-2}\right) \right\}^{(1)} = \psi(\alpha_\sigma) - \log(\beta_\sigma).
\end{aligned}$$

The variational distribution of $\boldsymbol{\Omega}^{(a)}$ is not tractable, but the conditional distribution of its last column is tractable [Wang, 2015], allowing for conditional maximisation of the last column while keeping the rest of the matrix entries fixed,

$$\begin{aligned}
\boldsymbol{\Omega}^{(a)}_{-ii} &= \left(\boldsymbol{\Omega}^{(a)}_{i-i}\right)^T = -\left\{ (\mathbf{S}^{(a)}_{ii} + \lambda) \left(\boldsymbol{\Omega}^{(a)}_{-i-i}\right)^{-1} + \text{diag}\left(\mathbf{D}^{(a)}_{-ii}\right) \right\}^{-1} \mathbf{S}^{(a)}_{-ii}, \\
\boldsymbol{\Omega}^{(a)}_{ii} &= \boldsymbol{\Omega}^{(a)}_{i-i} \left(\boldsymbol{\Omega}^{(a)}_{-i-i}\right)^{-1} \boldsymbol{\Omega}^{(a)}_{-ii} + \frac{N_a}{\mathbf{S}^{(a)}_{ii} + \lambda},
\end{aligned}$$

where $\mathbf{S}^{(a)}$ is the sample covariance of network (a), subscript $-i$ refers to the removal of ith row (the first subscript) or column (the second subscript), and $\mathbf{D}^{(a)}$ is a $P \times P$ matrix with entries

$$\mathbf{D}^{(a)}_{ij} = \frac{(\delta^{(a)}_{ij})^{(1)}}{\nu_1^2} + \frac{1 - (\delta^{(a)}_{ij})^{(1)}}{\nu_0^2}.$$

1.1.1 Maintaining positive definiteness in the precision matrix

With these updates, the positive definiteness constraint for $\boldsymbol{\Omega}^{(a)}$ is maintained at each iteration as long as the initial value is positive definite. This can be shown with a proof-by-induction type argument equivalent to that of Wang [2012]:

- If the initial $\boldsymbol{\Omega}^{(a)}$ is positive definite, then each of its P leading principal minors (LPMs), which are the determinants of each upper left corner of the matrix, are positive. The model is such that this is always true.
- Let $\boldsymbol{\Omega}^{(a)}$ be the updated $\boldsymbol{\Omega}^{(a)}$ after a single iteration that updates the last row and column. Each of the LPMs are the same as in $\boldsymbol{\Omega}^{(a)}$ except the last one (of order P).
- The last LPM is equal to the determinant of the whole matrix. It can be shown that

$$\det(\boldsymbol{\Omega}^{(a)}_\star) = \eta \det(\boldsymbol{\Omega}^{(a)}_{(-p)(-p)}) = \eta LPM_{(p-1)} \tag{1}$$

where $LPM_{(p-1)}$ is the $(p-1)^{\text{th}}$ LPM of $\mathbf{\Omega}^{(a)}$ and thus positive. The η variable is expressed as

$$\eta = \mathbf{\Omega}_{pp} - \mathbf{\Omega}_{(-p)p}^{T}\mathbf{\Omega}_{(-p)(-p)}^{-1}\mathbf{\Omega}_{(-p)p} > 0, \tag{2}$$

and thus also positive.

- Therefore, $\det(\mathbf{\Omega}_{\star}^{(a)}) > 0$ and so the updated $\mathbf{\Omega}_{\star}^{(a)}$ is positive definite.
- By induction, positive definiteness is thus maintained for all following updates of $\mathbf{\Omega}_{\star}^{(a)}$.

1.2 Evidence Lower Bounds (ELBOs)

The VBECM algorithm is implicitly maximising the Evidence Lower Bound of both the complete variational distribution and of all of its sub-distributions. These are given as:

$$\mathcal{L}(q) = \sum_{a\in\mathcal{A}} \mathcal{L}_{\boldsymbol{Y}^{(a)}}(\boldsymbol{Y}^{(a)} \mid \mathbf{\Omega}^{(a)}) \quad + \quad \sum_{a\in\mathcal{A}} \mathcal{L}_{\mathbf{\Omega}^{(a)}}(\mathbf{\Omega}^{(a)} \mid \{\delta_{ij}^{(a)}\}_{i<j}) + \sum_{a\in\mathcal{A}}\sum_{i<j} \mathcal{L}_{\delta_{ij}^{(a)}, z_{ij}^{(a)}}(\delta_{ij}^{(a)}, z_{ij}^{(a)} \mid \zeta_{ij}, \beta_{ij})$$
$$+\mathcal{L}_{\zeta_{ij}}(\zeta_{ij}) + \sum_{i<j} \mathcal{L}_{\beta_{ij}}(\beta_{ij} \mid \sigma^2) + \mathcal{L}_{\sigma^2}(\sigma^2),$$

where

$$\begin{aligned}
\mathcal{L}_{\boldsymbol{Y}^{(a)}}(\boldsymbol{Y}^{(a)} \mid \mathbf{\Omega}^{(a)}) &= \log p(\boldsymbol{Y}^{(a)} \mid \mathbf{\Omega}^{(a)}) = \frac{N_a}{2}\log \mid \mathbf{\Omega}^{(a)} \mid -\frac{1}{2}\mathrm{tr}(\boldsymbol{Y}^{(a)\top}\boldsymbol{Y}^{(a)}\mathbf{\Omega}^{(a)}), \\
\mathcal{L}_{\mathbf{\Omega}^{(a)}}(\mathbf{\Omega}^{(a)} \mid \delta_{ij}) &= \mathbb{E}_{q(\mathbf{\Omega},\mathbf{\Theta})}\left\{ \sum_{i=1}^{P} \log p(\mathbf{\Omega}_{ii}^{(a)}) + \sum_{i<j} \log p(\mathbf{\Omega}_{ij}^{(a)} \mid \delta_{ij}^{(a)}) \right\} \\
&= -\frac{\lambda}{2}\sum_{i=1}^{P} \mathbf{\Omega}_{ii}^{(a)} - \log \nu_1 \sum_{i<j} (\delta_{ij}^{(a)})^{(1)} - \log \nu_0 \sum_{i<j} \left(1 - (\delta_{ij}^{(a)})^{(1)}\right) \\
&\quad -\frac{1}{2}\sum_{i<j}(\mathbf{\Omega}_{ij}^{(a)})^2 \left(\frac{(\delta_{ij}^{(a)})^{(1)}}{\nu_1^2} + \frac{1-(\delta_{ij}^{(a)})^{(1)}}{\nu_0^2} \right), \\
\mathcal{L}_{\delta_{ij}^{(a)}, z_{ij}^{(a)}}(\delta_{ij}^{(a)}, z_{ij}^{(a)} \mid \{\zeta_{ij}\}_{i<j}, \{\beta_{ij}\}_{i<j}) &= \mathbb{E}_{q(\mathbf{\Omega},\mathbf{\Theta})}\left\{ \log p(\delta_{ij}^{(a)} \mid z_{ij}^{(a)}) + \log p(z_{ij}^{(a)} \mid \{\zeta_{ij}\}_{i<j}, \{\beta_{ij}\}_{i<j}) \right\} \\
&\quad -\mathbb{E}_{q(\mathbf{\Omega},\mathbf{\Theta})}\left\{ \log q(z_{ij}^{(a)} \mid \delta_{ij}^{(a)}) - \log q(\delta_{ij}^{(a)}) \right\} \\
&= \left[\delta_{ij}^{(a)} \log \mathbb{1}\{z_{ij}^{(a)} \geq 0\}\right]^{(1)} + \left[\left(1-\delta_{ij}^{(a)}\right) \log \mathbb{1}\{z_{ij}^{(a)} < 0\}\right]^{(1)} \\
&\quad -\frac{1}{2}\left((z_{ij}^{(a)})^{(2)} - 2(\alpha_{ij}^{(a)})^{(1)}(z_{ij}^{(a)})^{(1)} + (\alpha_{ij}^{(a)})^{(2)} \right) \\
&\quad -\mathbb{E}_{q(\mathbf{\Omega},\mathbf{\Theta})} \log q(z_{ij}^{(a)} \mid \delta_{ij}^{(a)}) - \mathbb{E}_{q(\mathbf{\Omega},\mathbf{\Theta})} \log q(\delta_{ij}^{(a)}),
\end{aligned}$$

with

$$\begin{aligned}
\mathbb{E}_{q(\mathbf{\Omega},\mathbf{\Theta})} \log q(z_{ij}^{(a)} \mid \delta_{ij}^{(a)}) &= -\frac{1}{2}\left\{ (z_{ij}^{(a)})^{(2)} - 2(z_{ij}^{(a)})^{(1)}(\alpha_{ij}^{(a)})^{(1)} + \left((\alpha_{ij}^{(a)})^{(1)}\right)^2 \right\} \\
&\quad -(\delta_{ij}^{(a)})^{(1)} \log\left\{ \Phi\left((\alpha_{ij}^{(a)})^{(1)}\right) \right\} - \left(1-(\delta_{ij}^{(a)})^{(1)}\right) \log\left\{ 1 - \Phi\left((\alpha_{ij}^{(a)})^{(1)}\right) \right\} \\
&\quad +(\delta_{ij}^{(a)})^{(1)} \left[\log \mathbb{1}\{z_{ij}^{(a)} > 0\}\right]^{(1)} + \left(1-(\delta_{ij}^{(a)})^{(1)}\right)\left[\log \mathbb{1}\{z_{ij}^{(a)} \leq 0\}\right]^{(1)},
\end{aligned}$$

and

$$\mathbb{E}_{q(\mathbf{\Omega},\mathbf{\Theta})} \log q(\delta_{ij}^{(a)}) = (\delta_{ij}^{(a)})^{(1)} \log\left((\delta_{ij}^{(a)})^{(1)}\right) + \left(1-(\delta_{ij}^{(a)})^{(1)}\right) \log\left(1-(\delta_{ij}^{(a)})^{(1)}\right),$$

which is further simplified to

$$
\begin{aligned}
\mathcal{L}_{\delta_{ij}^{(a)}, z_{ij}^{(a)}}(\delta_{ij}^{(a)}, z_{ij}^{(a)} \mid \{\zeta_{ij}\}_{i<j}, \{\beta_{ij}\}_{i<j}) \quad = \quad & -\frac{1}{2}(\alpha_{ij}^{(a)})^{(2)} + \frac{1}{2}\left((\alpha_{ij}^{(a)})^{(1)}\right)^2 \\
& +(\delta_{ij}^{(a)})^{(1)} \log\left\{\Phi\left((\alpha_{ij}^{(a)})^{(1)}\right)\right\} + \left(1-(\delta_{ij}^{(a)})^{(1)}\right)\log\left\{1-\Phi\left((\alpha_{ij}^{(a)})^{(1)}\right)\right\} \\
& -(\delta_{ij}^{(a)})^{(1)} \log\left((\delta_{ij}^{(a)})^{(1)}\right) - \left(1-(\delta_{ij}^{(a)})^{(1)}\right)\log\left(1-(\delta_{ij}^{(a)})^{(1)}\right).
\end{aligned}
$$

In addition, we have

$$
\begin{aligned}
\mathcal{L}_{\zeta_{ij}}(\zeta_{ij}) &= \mathbb{E}_{q(\boldsymbol{\Omega},\boldsymbol{\Theta})}\Big\{\log p(\zeta_{ij}) - \log q(\zeta_{ij})\Big\} \\
&= -\frac{\sigma_{\zeta_{ij}}^2}{2t_0^2} + \frac{n_0\mu_{\zeta_{ij}}}{t_0^2} + \frac{1}{2}\Big\{1+\log(2\pi\sigma_{\zeta_{ij}}^2)\Big\}, \\
\mathcal{L}_{\beta_{ij}}(\beta_{ij} \mid \sigma^2) &= \mathbb{E}_{q(\boldsymbol{\Omega},\boldsymbol{\Theta})}\Big\{\log p(\beta_{ij} \mid \sigma^2) - \log q(\beta_{ij})\Big\} \\
&= \frac{1}{2}\left(\log\sigma^{-2}\right)^{(1)} - \frac{\sigma_{\beta_{ij}}^2}{2}\left(\sigma^{-2}\right)^{(1)} + \frac{1}{2}\Big\{1+\log\left(2\pi\sigma_{\beta,ij}^2\right)\Big\}, \\
\mathcal{L}_{\sigma}(\sigma^{-2}) &= \mathbb{E}_{q(\boldsymbol{\Omega},\boldsymbol{\Theta})}\Big\{\log p(\sigma^{-2}) - \log q(\sigma^{-2})\Big\} \\
&= (a_\sigma - 1)\left(\log\sigma^{-2}\right)^{(1)} - b_\sigma\left(\sigma^{-2}\right)^{(1)} \\
&\quad -\alpha_\sigma \log\beta_\sigma + \log\Gamma(\alpha_\sigma) - (\alpha_\sigma - 1)\left(\log\sigma^{-2}\right)^{(1)} + \beta_\sigma\left(\sigma^{-2}\right)^{(1)} \\
&= (a_\sigma - \alpha_\sigma)\left(\log\sigma^{-2}\right)^{(1)} - (b_\sigma - \beta_\sigma)\left(\sigma^{-2}\right)^{(1)} - \alpha_\sigma\log\beta_\sigma + \log\Gamma(\alpha_\sigma),
\end{aligned}
$$

where $B(a,b) = \Gamma(a)\Gamma(b)/\Gamma(a+b)$ is the beta function. In addition, we have

$$
\begin{aligned}
\left((\alpha_{ij}^{(a)})^{(1)}\right)^2 - (\alpha_{ij}^{(a)})^{(2)} &= \left(\mu_{\zeta_{ij}} + \mu_{\beta_{ij}}a\right)^2 - \left(\sigma_{\zeta_{ij}}^2 + 2\mu_{\zeta_{ij}}\mu_{\beta_{ij}}a + \sigma_{\beta_{ij}}^2 a^2\right) \\
&= \mu_{\zeta_{ij}}^2 - \sigma_{\zeta_{ij}}^2 + a^2\left\{\left(\mu_{\beta_{ij}}\right)^2 - \sigma_{\beta_{ij}}^2\right\}.
\end{aligned}
$$

2 Addendum to simulation experiments

2.1 Simulating positive-definite precision matrices with appropriate sample-level heterogeneity

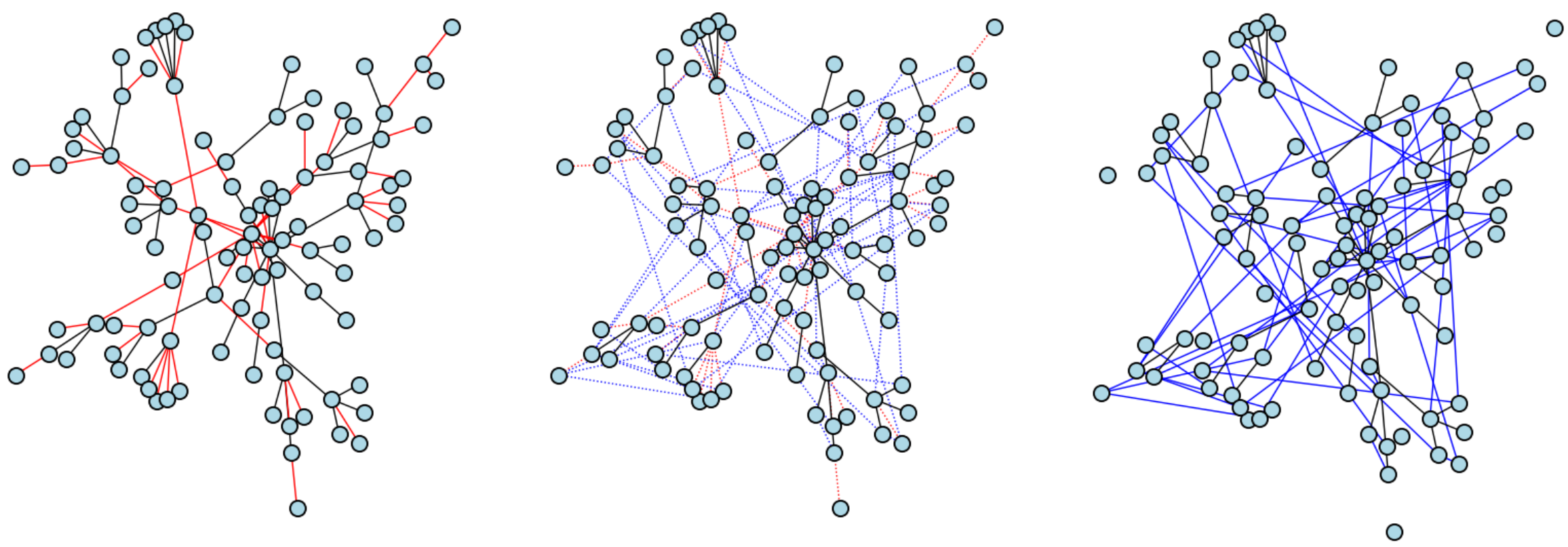

Supplementary Figure S1: Example simulated conditional dependence networks used in the main simulation. Black edges remain constant across all networks. Red edges represent connections that disappear as the covariate increases, while blue edges indicate new connections that appear. The left panel shows the initial scale-free network ($a = 1$). The middle panel represents the transition phase of networks ($a = 2, 3$), where edge strengths vary as edges appear or disappear. The right panel shows network ($a = 4$), where disappearing edges are absent (zero-valued), and appearing edges have reached their maximum signal strength.

To generate the set of precision matrices, $\{\mathbf{\Omega}^{(a)}\}_{a\in\mathcal{A}}$, we begin by generating $\mathbf{\Omega}^{(1)}$ such that it is a $P \times P$ positive definite matrix that represents a scale-free network. This results in $P-1$ edges in the first network. Half of the edges are then randomly sampled to "disappear" as the covariate increases. The same number of non-edges are sampled to "appear" as the covariate increases. In this case, an edge "disappearing" means that its corresponding partial correlation linearly decreases as the covariate increases and equal to zero (a non-edge) in the network corresponding to the largest covariate value. "Appearing" edges similarly linearly increase from zero to an appropriate value in the network corresponding to the largest covariate value. Edges that neither appear nor disappear stay uniform across networks (homogenous edges). The precision matrix entry $\mathbf{\Omega}_{ij}^{(a)}$, where $1 \leq i < j \leq P$ and $a \in \{1, ..., A\}$, follows

$$
\begin{aligned}
\mathbf{\Omega}_{ij}^{(a)} &= \left(\frac{A-a}{A-1}\right) \cdot \mathbf{\Omega}_{ij}^{(1)} \quad \text{when } (i,j) \in E_-, \\
\mathbf{\Omega}_{ij}^{(a)} &= \eta \cdot \left(\frac{a-1}{A-1}\right) \cdot \omega \quad \text{when } (i,j) \in E_+, \\
\mathbf{\Omega}_{ij}^{(a)} &= \mathbf{\Omega}_{ij}^{(1)} \quad \text{when } (i,j) \notin E_- \cup E_+
\end{aligned}
$$

where E_- is the set of disappearing edges, E_+ is the set of appearing edges, η is sampled from $\{-1, 1\}$ with equal probability and ω is a value set equal to the maximum absolute non-zero value found in $\mathbf{\Omega}^{(1)}$, i.e., $\omega = \mid \max_{1\leq i<j\leq P} (\mathbf{\Omega}_{ij}^{(1)}) \mid$. As such, the magnitude of precision matrix entries increase linearly for appearing edges to a value appropriate to the setting, where η decides the sign of partial correlations, decrease linearly for disappearing edges such that the edge is not present in the final network, and remain constant for homogenous edges.

The simulated precision matrices are not necessarily positive definite and therefore we repeat the algorithm with different seeds until positive definite matrices are found. The networks generated by this algorithm are shown schematically in Figure S1.

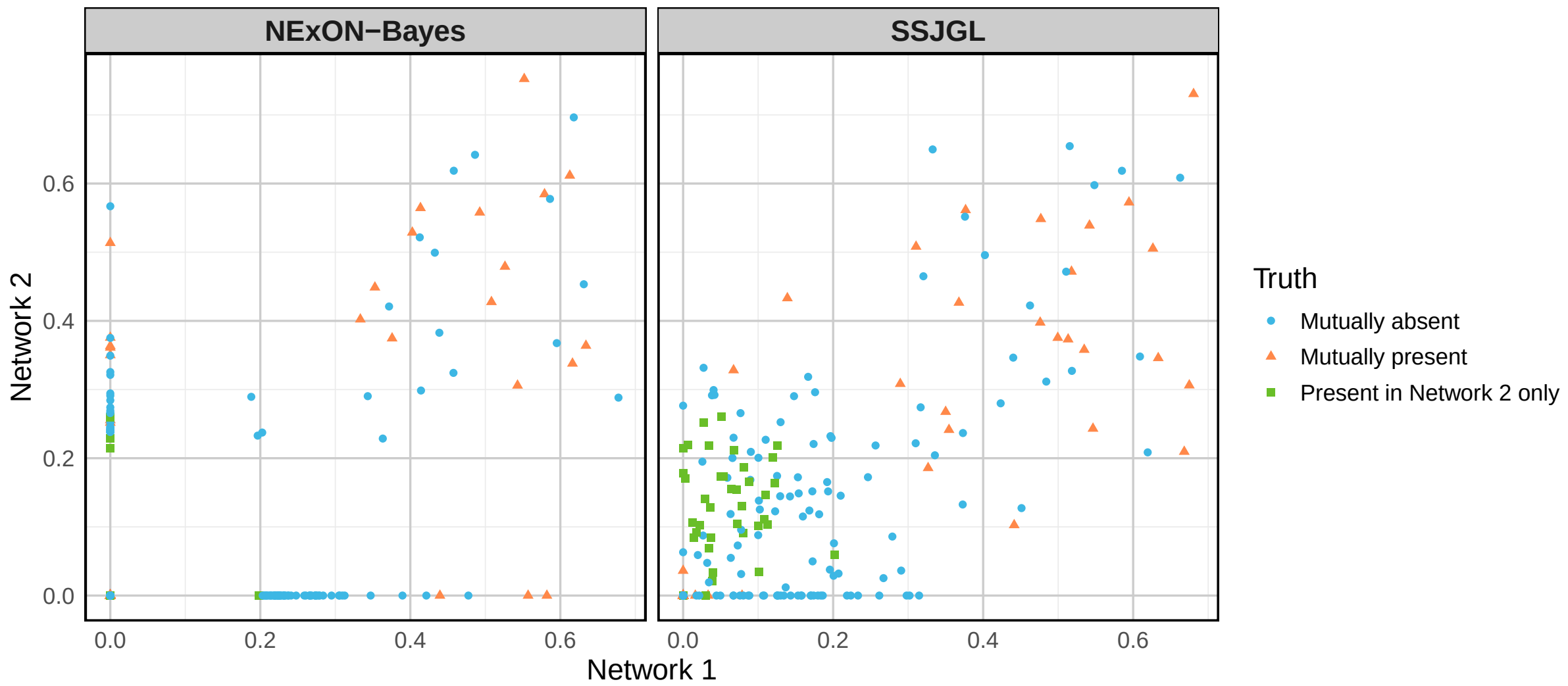

Supplementary Figure S2: Comparison of estimated partial correlations for network (2) against network (1) using NExON-Bayes and SSJGL (one replicate). SSJGL rarely predicts an edge in network 2 without making the same prediction in network 1, leading to frequent errors. Note that network (1) is a subset of network (2) and so there are no entries that solely belong to network (1)

2.2 Further analysis of NExON-Bayes' ability to distinguish heteregenous samples

Whilst calculating the precision and recall of the graphical estimations made by each model serves as a well-rounded assessment of their ability to recover conditional independence structures, it is also important in this scenario to analyse how well the models are able to encapsulate sample-level heterogeneity.

Figure S2 compares estimated partial correlations of networks (1) and (2) of the main simulation. SSJGL rarely estimates edges unique to network (2) well, because SSJGL infers similarity structure across networks, rather than modelling a covariate-dependent correlation. On the other hand, NExON-Bayes is far better at making positive predictions for one network and not the other.

2.3 Full tabulated results of the main simulation

Table S1 shows the results of the main simulation. The metrics used are accuracy, Matthew's correlation coefficient (MCC), precision, recall and estimated sparsity. Precision and recall are the principal metrics used in the main text. Precision measures the proportion of predicted positive cases that are correct, whilst recall measures the proportion of actual positive cases that are correctly identified. They are calculated using

$$\text{Precision} = \frac{TP}{TP+FP};$$
$$\text{Recall} = \frac{TP}{TP+FN}.$$

MCC measures how well the predicted labels correlate with the true labels and considers all four outcomes of the confusion matrix, capturing the balance between each class (positive/negative). It is expressed as

$$\text{MCC} = \frac{(TP \times TN) - (FP \times FN)}{\sqrt{(TP+FP)(TP+FN)(TN+FP)(TN+FN)}}.$$

We also calculate accuracy, which gives the proportion of edges that are correctly classified over all edges. In a sparse setting, accuracy is expected to be very high (close to 1) as true negatives dominate predictions. We calculate accuracy as

$$\text{Accuracy} = \frac{TN+TP}{TN+FP+TP+FN}.$$

Estimated sparsity ($\hat{\boldsymbol{\rho}}_\mathbf{0}$) is the number of predicted edges over the total edges:

$$\hat{\boldsymbol{\rho}}_\mathbf{0} = \frac{TP+FP}{TP+FP+TN+FN}.$$

2.4 Simulations under misspecified settings

The studies performed in Section 3.2 and 3.3 of the main text use simulated data that is well specified to the framework of NExON-Bayes. To obtain a more complete metric of NExON-Bayes' performance and test its robustness, we here introduce three further simulation studies that, to a varying degree, deviate from the specification of the model. We assess the estimates made by several comparable and competing models where data is generated from normal distributions with (i): identical precision matrices, (ii): independently generated precision matrices and (iii): precision matrices with non-monotonic relationships between the covariate and the conditional dependence, which is a purposely *mis-specified* framework. Fifty replicates for each model in each scenario are performed using networks with $P = 50$. The precision, recall and MCC are averaged over all networks and shown in Table S2, where these mean values are presented along with standard deviations.
Comparison is made to the same models as in the main simulation (covdepGE, mGHS & SSJGL) as well as SSL [Li and McCormick, 2019, Xi and Ruffieux, 2024], which serves as a comparable single-network estimation model.
Scenario (i) corresponds to a setting in which the model is expected to recover a highly sparse association between covariates and conditional dependence, with most β_{ij} close to zero. In this scenario, NExON-Bayes attains the highest mean precision (0.903) and MCC (0.706). Scenario (ii) simulates noisy associations between covariates and conditional dependence, which weakens the statistical signals the model uses to estimate each β_{ij} and results in worse performance (precision = 0.865, recall = 0.465, MCC

Supplementary Table S1: Performance of each model (NExON-Bayes, SSJGL, covdepGE, mGHS) across four networks of 100 nodes. The given metrics are accuracy, MCC, precision, recall, and estimated sparsity ($\hat{\boldsymbol{\rho}}_{\mathbf{0}}$). Values are means across 100 replicates with standard deviations in parentheses. The best performance in each metric for each network is written in bold.

**Model**	**Net.**	**Accuracy**	**MCC**	**Precision**	**Recall**	$\hat{\boldsymbol{\rho}}_{\mathbf{0}}$
NExON-Bayes	1	**0.984** (0.002)	**0.558** (0.044)	**0.642** (0.083)	0.501 (0.039)	0.016 (0.002)
	2	0.975 (0.001)	0.458 (0.030)	0.692 (0.068)	0.320 (0.034)	0.014 (0.002)
	3	**0.980** (0.001)	**0.608** (0.025)	0.778 (0.058)	0.490 (0.030)	0.019 (0.002)
	4	**0.988** (0.002)	**0.725** (0.041)	0.697 (0.077)	0.769 (0.031)	0.022 (0.003)
SSJGL	1	0.980 (0.001)	0.409 (0.029)	0.505 (0.048)	0.347 (0.026)	0.024 (0.001)
	2	**0.980** (0.001)	**0.592** (0.024)	**0.844** (0.046)	0.427 (0.018)	0.025 (0.001)
	3	**0.980** (0.001)	0.584 (0.023)	**0.836** (0.047)	0.420 (0.017)	0.025 (0.001)
	4	0.986 (0.001)	0.608 (0.025)	0.716 (0.046)	0.528 (0.016)	0.025 (0.001)
covdepGE	1	0.979 (0.002)	0.208 (0.081)	0.436 (0.165)	0.109 (0.047)	0.005 (0.001)
	2	0.969 (0.001)	0.140 (0.024)	0.429 (0.064)	0.052 (0.017)	0.004 (0.001)
	3	0.972 (0.001)	0.293 (0.042)	0.705 (0.080)	0.129 (0.024)	0.005 (0.001)
	4	0.982 (0.000)	0.453 (0.025)	0.578 (0.016)	0.370 (0.038)	0.013 (0.001)
mGHS	1	0.945 (0.013)	0.447 (0.038)	0.254 (0.044)	**0.854** (0.044)	0.069 (0.014)
	2	0.950 (0.010)	0.510 (0.025)	0.362 (0.049)	**0.785** (0.057)	0.067 (0.013)
	3	0.951 (0.010)	0.494 (0.036)	0.361 (0.055)	**0.738** (0.053)	0.063 (0.012)
	4	0.955 (0.013)	0.538 (0.057)	0.317 (0.067)	**0.969** (0.021)	0.064 (0.013)

= 0.624) than in scenario (i). Scenario (iii) is a misspecified framework that simulates a non-monotonic association between covariates and conditional dependence. It should also be noted that this scenario is also more difficult due to weaker signals in intermediary networks (2) & (4) (an artefact discussed in Section 3.3 of the main text). The estimates made by NExON-Bayes are worse than in any other scenario, and whilst a high precision is maintained (0.810), NExON-Bayes becomes inferior to other models, suffering with a poor recall (0.385) and low MCC (0.545).

Results for the other models are interesting too. covdepGE performs similarly to NExON-Bayes in scenarios (i) and (ii), improving greatly in a lower dimension ($P = 50$) compared to the main simulation (where $P = 100$). This is likely due to covdepGE tending towards sparser estimates in higher dimensions. For the same reason, covdepGE struggles in scenario (iii) where there are more networks.

SSL performs marginally worse than NExON-Bayes in all metrics, which is consistent with the AUC study performed in Section 3.2 of the main text. Whilst we may expect SSL to outperform NExON-Bayes in scenario (iii), where NExON-Bayes struggles to discern the non-monotonic relation between covariate and conditional dependence, the weaker signals of some of these networks means that, without making a joint estimation and sharing information across networks, SSL also struggles in this scenario. This is also consistent with the results in Section 3.2.

mGHS consistently estimates networks with very high recall ((i) 0.970, (ii) 0.826 & (iii) 0.842) and mid-range precision ((i) 0.475, (ii) 0.349 & (iii) 0.443), outperforming all models in terms of recall and MCC (0.587) in scenario (iii). mGHS treats covariates exchangeably and thus can deal with non-monotonic relations where NExON-Bayes struggles. This further supports NExON-Bayes inapplicability in settings where the relation between covariate and conditional dependence is known to vary non-monotonically. In these cases, models that either treat networks exchangeably (like mGHS and SSJGL), or those that can make more flexible estimations of the relation between covariate and conditional dependence (covdepGE) are recommended for use over NExON-Bayes.

Overall, these results show that NExON-Bayes can still perform well compared to other similar joint

Supplementary Table S2: Performance of each model (NExON-Bayes, SSJGL, SSL, covdepGE, mGHS) across performance metrics (MCC, precision, recall) for models estimating networks under three data-generating settings. Reported values are means with standard errors in parentheses from 100 replicates. Unless otherwise noted, $P = 50$ variables, $|A| = 3$ networks, and $N = 100$ samples are generated from each corresponding precision matrix. Best performance for each metric within each panel is in bold.

Model	MCC	Precision	Recall
**(i) Data generated from identical precision matrices**			
**NExON-Bayes**	**0.706 (0.003)**	**0.903 (0.004)**	0.566 (0.004)
**SSJGL**	0.450 (0.004)	0.299 (0.003)	0.945 (0.005)
**SSL**	0.666 (0.003)	0.889 (0.004)	0.515 (0.004)
**covdepGE**	0.426 (0.009)	0.892 (0.013)	0.229 (0.007)
**mGHS**	0.661 (0.005)	0.475 (0.007)	**0.970 (0.004)**
**(ii) Data generated from independent precision matrices**			
**NExON-Bayes**	**0.624 (0.003)**	**0.865 (0.005)**	0.465 (0.004)
**SSJGL**	0.338 (0.002)	0.174 (0.001)	**0.857 (0.003)**
**SSL**	0.621 (0.003)	0.863 (0.005)	0.463 (0.004)
**covdepGE**	0.427 (0.006)	0.835 (0.007)	0.232 (0.006)
**mGHS**	0.508 (0.006)	0.349 (0.007)	0.826 (0.005)
**(iii) Data generated from precision matrices with non-monotonic correlations** ($|A| = 5$)			
**NExON-Bayes**	0.545 (0.003)	**0.810 (0.006)**	0.385 (0.003)
**SSJGL**	0.467 (0.003)	0.478 (0.005)	0.506 (0.002)
**SSL**	0.514 (0.003)	0.761 (0.007)	0.368 (0.003)
**covdepGE**	0.278 (0.003)	0.599 (0.005)	0.143 (0.002)
**mGHS**	**0.587 (0.003)**	0.443 (0.004)	**0.842 (0.004)**

(and single) network estimation models in scenarios less suited to it, although caution should be taken in settings contextually suspected to be mis-specified.

2.5 Computational runtimes

We next evaluate the computational runtimes of each model used in simulations by analysing how long each model takes to make an estimation across a range of network sizes ($10 \leq P \leq 250$) and network quantity ($|\mathcal{A}| = 2, 4, 6, 8$). A time limit of 10^5 seconds (≈ 28 hours) is enforced in this simulation, which aligns with the default maximum time limit of 1.3×10^5 (36 hours) in place on the CPU cluster on which the simulations were performed [University of Cambridge Research Computing Services, 2025]. Overall, we recommend using NExON-Bayes in scenarios where $10 \leq P \leq 250$, exercising caution above this range, especially when the number of networks is large. Within this range, NExON-Bayes performs inference faster than the other joint models tested (covdepGE, mGHS, SSJGL). The runtime of NexON-Bayes appears to scale approximately linearly with the number of networks, which is to be expected. Figure S3 shows that NExON-Bayes has a similar (if not marginally slower) runtime in all scenarios compared to SSL, which is expected due to their similar (deterministic) VBECM inference algorithms. Furthermore, NExON-Bayes has a faster computational runtime than SSJGL and covdepGE in all settings. covdepGE scales well up to $P = 150$, but above this value has a tendency to converge to empty precision matrix estimations and so we deem it generally inapplicable to settings with these dimensions. We also found mGHS to be inapplicable to settings with $P > 200$ due to bad performance, suggesting a lack of convergence. Within the range $50 < P < 200$ mGHS does scale impressively. The impressive scalability of mGHS may be attributable to the fact that the maximum number of iterations in the stochastic sampling algorithm employed by mGHS is fixed across all experimental scenarios, following the implementation choices in Busatto and Stingo [2025]. This design choice explains the nearly constant runtimes observed for mGHS as the quantity of networks increases and raises questions of whether convergence is reached in the higher-dimensional settings. This is backed up by our simulations, which show that whilst mGHS performs well with $P = 50$, performance is seriously hampered even

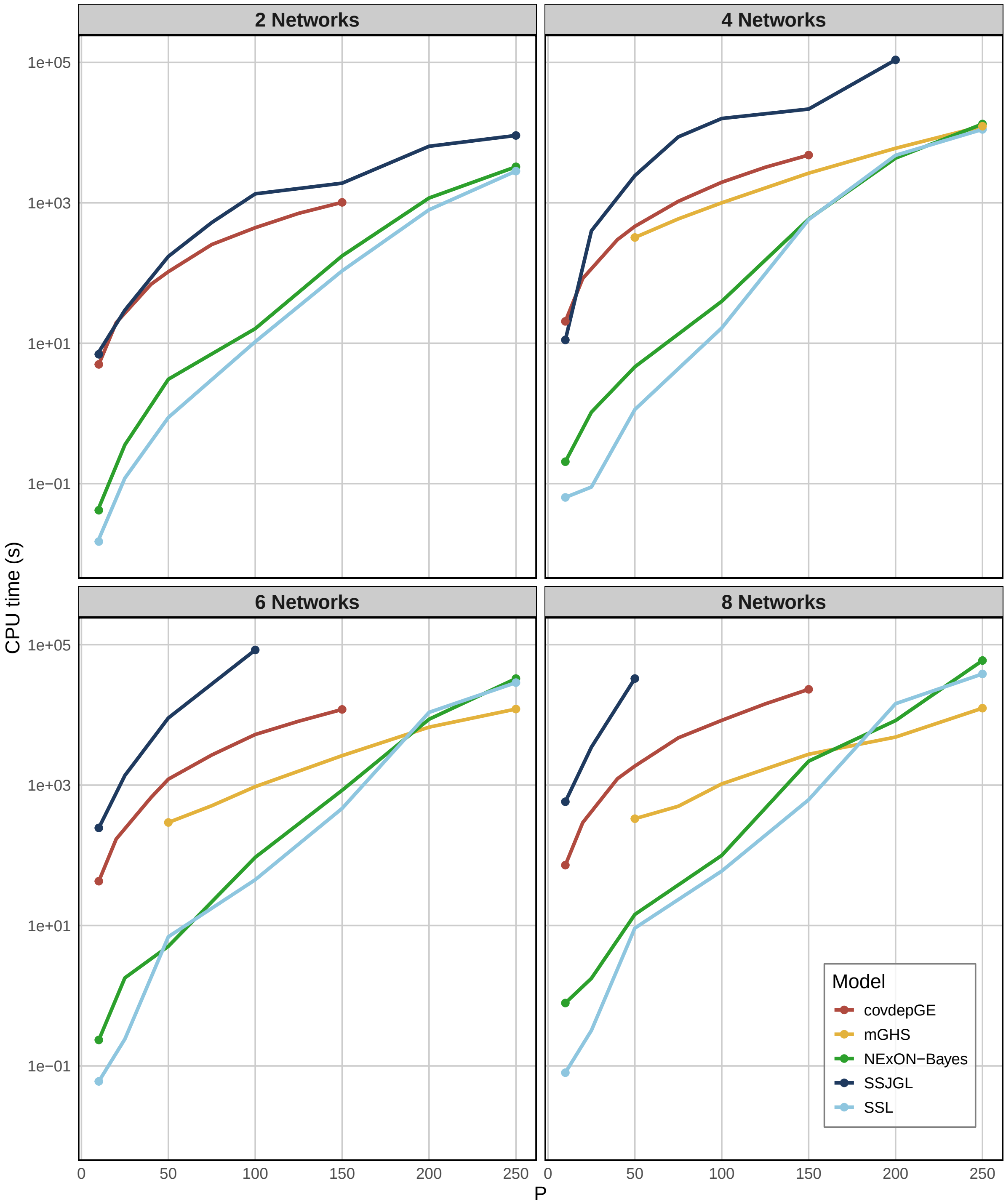

Supplementary Figure S3: Computational runtimes in seconds on a logarithmic scale for covdepGE, mGHS, NExON-Bayes, SSJGL, and SSL across increasing numbers of networks ($|\mathcal{A}| = 2, 4, 6, 8$) and a range dimensions ($10 \leq P \leq 250$). In this evaluation, code is run through a CPU cluster on a high performance computing (HPC) service [University of Cambridge Research Computing Services, 2025], which has a default maximum time limit of 36 hours (1.3e+05 seconds).

at $P = 100$. As such, the results of this runtime simulation should be interpreted with caution. The missing mGHS results for $P < 50$ and for the 2-network setting arise because the Gibbs sampler it employs frequently failed to mix adequately, getting 'stuck' and preventing the model from producing a valid network estimate. Overall, these results are promising as NExON-Bayes is applicable to and will perform estimation more quickly than most comparitive models in many practically relevant network sizes found in the literature. For example, Ni et al. [2022] estimate the networks of patients whose data was collected by the Multiple Myeloma Research Consortium containing $P = 33$ gene expressions per patient, Xi and Ruffieux [2024] explore a dataset with $P = 137$ genetic and monocyte expression variables, Chen et al. [2025] explore the same TCGA dataset as is explored in this work that has $P = 131$ proteomic variables.

2.5.1 Models restricted by runtime

Two covariate-dependent graphical models that would be well worth consideration and comparison to NExON-Bayes are 1) the 'Gaussian Graphical models with covariates' (GGMx) [Ni et al., 2022], which incorporates both a strength and a sparsity pattern between precision matrices and covariates and 2) the partition based graphical model (PxG) [Niu et al., 2024] which uses partition models that adapt graph structure based on similarity in covariates. The methodological aspects of these works are extremely interesting, however we found the computational runtimes of both models to exceed practical consideration for scenarios explored in our work, and have thus excluded them from simulations and major discussions. Both corresponding papers acknowledge poor applicability to such high-dimensional settings. Figure S4 shows the runtimes for settings where $|\mathcal{A}| = 4$ and $P < 50$. Both models have similar runtimes, although PxG fails for $P > 20$. They are both approximately 2 orders of magnitude slower than SSJGL (which, referencing Figure S3, is shown to scale poorly) and approximately 4 orders of magnitude slower than NExON-Bayes.

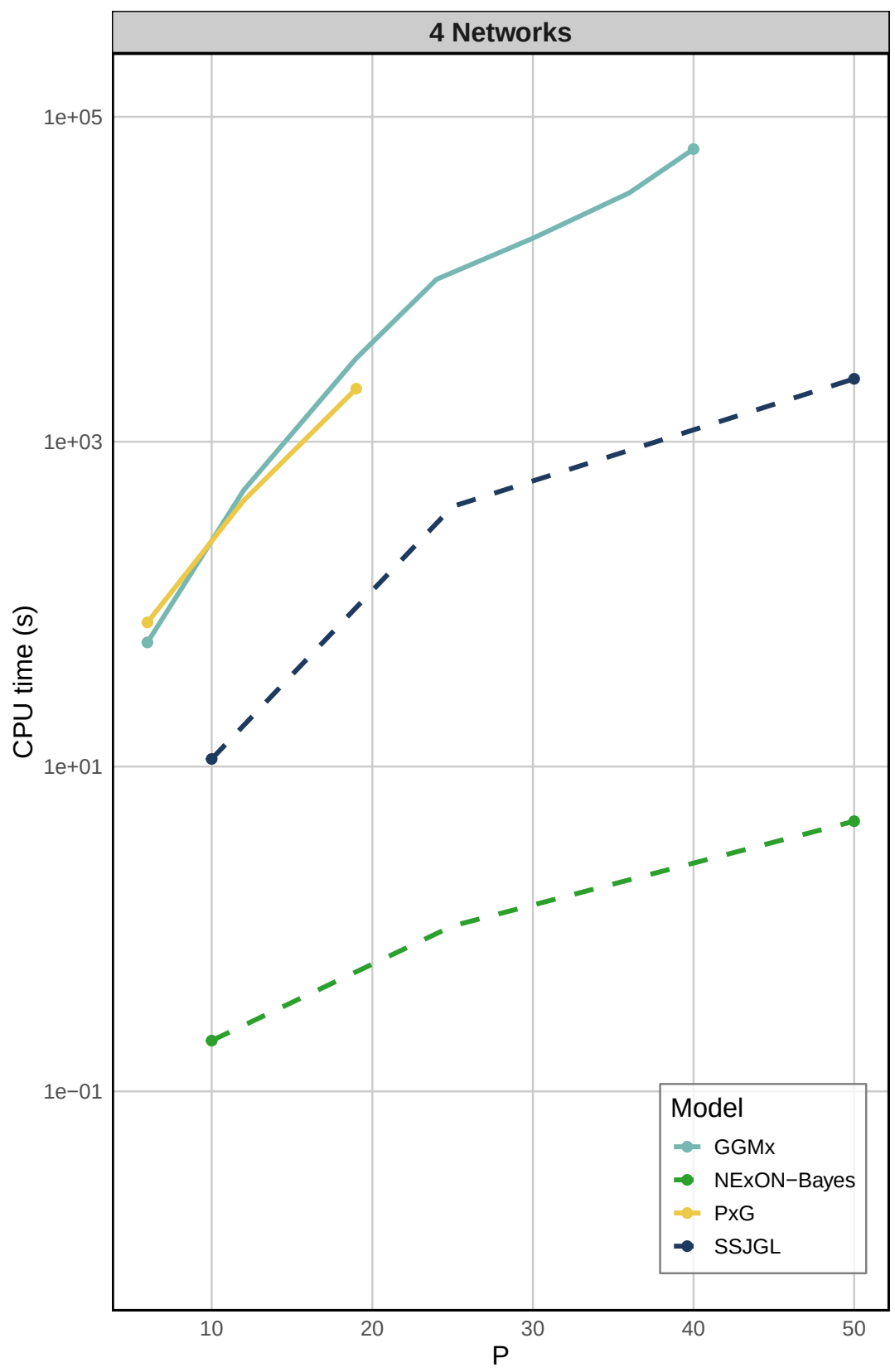

Supplementary Figure S4: Computational runtimes in seconds on a logarithmic scale for GGMx, PxG, NExON-Bayes and SSJGL in settings with 4 networks ($|\mathcal{A}| = 4$) and a range of dimensions ($10 \leq P \leq 50$). We constrain results to an approximate upper limit of $\approx$ 36 hours (1.3e+05 seconds), which is the standard upper limit for jobtime on the High Performance Computing (HPC) service that we were using. NExON-Bayes and SSJGL are included here for comparison- see Figure S3 for full results.

3 Addendum to BRCA application

3.1 Evidence found in STRING database

The STRING (*Search Tool for the Retrieval of Interacting Genes/Proteins*) database is a comprehensive resource of known and predicted protein-protein interactions, integrating data from experiments, computational prediction methods, and public databases to support functional analysis of gene sets [Szklarczyk et al., 2023]. To further assess whether NExON-Bayes provides a more complete estimation of the proteomic networks of BRCA patients at different stages than the vanilla model SSL, we here find the percentage of edges that are predicted by each model that are also found in the STRING database. In particular, we only consider protein-protein interactions that have been experimentally validated to a 90% confidence threshold. It should also be noted that the protein-protein interactions within this data set have been compiled for a *general* cancer setting, not just in *breast* cancer settings. Table S3 shows the results of this validation, where NExON-Bayes matches or outperforms SSL in all three networks.

Supplementary Table S3: Percentage of edges estimated by SSL and NExON-Bayes in the STRING dataset. The highest percentage of edges with evidence is in bold.

BRCA Stage	SSL	NExON-Bayes
I	3.1%	**3.2%**
II	**2.0%**	**2.0%**
III	2.0%	**2.3%**

3.2 Hub Proteins

Supplementary Table S4: The proteins with node degree larger than the 90th percentile in each network estimated by NExON-Bayes. Each protein's node degree and the gene that encodes them are also shown. The proteins that have node degree in the upper 10% in all three tumour stages are marked in bold. The proteins that only have node degree in the upper 10% in only one tumour stage are marked in red.

Stage I			Stage II			Stage III		
Protein	Gene	Degree	Protein	Gene	Degree	Protein	Gene	Degree
**GATA3**	*GATA3*	30	**GATA3**	*GATA3*	40	**GATA3**	*GATA3*	31
**ERALPHA**	*ESR1*	20	**ERALPHA**	*ESR1*	35	**ERALPHA**	*ESR1*	19
COLLAGENVI	*COL6A1*	18	**FIBRONECTIN**	*FN1*	28	**CYCLINB1**	*CCNB1*	16
**CYCLINB1**	*CCNB1*	17	**S6**	*RPS6*	24	COLLAGENVI	*COL6A1*	16
**FIBRONECTIN**	*FN1*	16	HSP70	*HSPA1A*	24	**FIBRONECTIN**	*FN1*	15
**MAPKPT202Y204**	*MAPK1*	14	CHK1PS345	*CHEK1*	24	**S6**	*RPS6*	13
**S6**	*RPS6*	13	**CAVEOLIN1**	*CAV1*	23	**MAPKPT202Y204**	*MAPK1*	13
GSK3ALPHABETAPS21S9	*GSK3A*	11	PCNA	*PCNA*	22	**CAVEOLIN1**	*CAV1*	12
EEF2	*EEF2*	11	**MAPKPT202Y204**	*MAPK1*	22	INPP4B	*INPP4B*	11
BETACATENIN	*CTNNB1*	11	MRE11	*MRE11A*	21	VEGFR2	*KDR*	10
VEGFR2	*KDR*	10	**CYCLINB1**	*CCNB1*	21	CMYC	*MYC*	10
**CAVEOLIN1**	*CAV1*	10	CHK1	*CHEK1*	21	BETACATENIN	*CTNNB1*	10
BAK	*BAK1*	10	BID	*BID*	21	BAK	*BAK1*	10

3.3 Agreement of top proteins in ranked summary vectors

To assess whether there is agreement at the protein level of the β_{ij} estimations made by both NExON-Bayes and GraphR, we identify the prominent proteins for each and find the level of overlap between the two. To do this, we can use different ranking criterion, which pertain to variants of a weighted node degree for each protein. These criteria, for each protein i, are

1. $\sum_{j\in\mathcal{V}}|\beta_{ij}|$, which is the weighted node degree for the graph $\mathcal{G}_{\pm} = (\mathcal{V}, E_{\pm})$, $\mathcal{V} = \{1, ...P\}$, $E_{\pm} = \{(i, j) : \beta_{ij} \neq 0\}$.

2. $\sum_{j\in\mathcal{V}}|\beta_{ij}|\mathbb{1}(\beta_{ij} > 0)$,which is the weighted node degree for the graph $\mathcal{G}_{+} = (\mathcal{V}, E_{+})$, $\mathcal{V} = \{1, ...P\}$, $E_{+} = \{(i, j) : \beta_{ij} > 0\}$.

3. $\sum_{j\in\mathcal{V}}|\beta_{ij}|\mathbb{1}(\beta_{ij} < 0)$, which is the weighted node degree for the graph which is the weighted node degree for the graph $\mathcal{G}_{-} = (\mathcal{V}, E_{-})$, $\mathcal{V} = \{1, ...P\}$, $E_{-} = \{(i, j) : \beta_{ij} < 0\}$.

Supplementary table S5 shows the results of Fisher's exact tests when each protein is ranked according to these criteria.

Supplementary Table S5: p-values from Fisher's exact tests of whether the overlap between proteins ranked above the kth percentile by ranking β_{ij} made by NExON-Bayes and GraphR exceeds chance, for $k \in \{80, 85, 90, 95\}$ and three ranking criteria. Bold entries denote significance at $\alpha = 0.05 \quad (5 \times 10^{-2})$.

Ranking criterion	95th	90th	85th	80th
1. $\sum_{j\in\mathcal{V}}\|\beta_{ij}\|$	$\mathbf{9.87 \times 10^{-5}}$	$\mathbf{2.03 \times 10^{-5}}$	$\mathbf{4.55 \times 10^{-4}}$	$\mathbf{2.69 \times 10^{-5}}$
2. $\sum_{j\in\mathcal{V}}\|\beta_{ij}\|\mathbb{1}(\beta_{ij} > 0)$	$\mathbf{3.04 \times 10^{-3}}$	1.22×10^{-1}	$\mathbf{1.48 \times 10^{-2}}$	$\mathbf{8.31 \times 10^{-3}}$
3. $\sum_{j\in\mathcal{V}}\|\beta_{ij}\|\mathbb{1}(\beta_{ij} < 0)$	$\mathbf{3.04 \times 10^{-3}}$	$\mathbf{3.32 \times 10^{-4}}$	$\mathbf{1.48 \times 10^{-2}}$	$\mathbf{8.31 \times 10^{-3}}$

3.4 Enriched gene sets found by NExON-Bayes and GraphR

The following tables list the gene sets found to be enriched in the $\boldsymbol{\beta}$ estimates of each model, ordered by size (how many genes in the set). A nominal p-value of 0.05 is used as a significance threshold.

Supplementary Table S6: Gene sets found to be enriched in the $\boldsymbol{\beta}_{\text{NExON}}$ network estimations made by NExON-Bayes to a significance of nominal p-value < 0.05 and FDR q-val < 0.25. Polarity indicates whether the gene set was found enriched in the positive correlative structure (+), the negative (−) or in both (±). Where gene sets are found enriched in both correlative structures, the nominal p-values are given as ' + , − ' . ⋆⋆ indicates a direct relation to apoptosis and ⋆ indicates an indirect relation.

**Gene Set**	**Size**	**nom. p-val**	**polarity**
WP_CHROMOSOMAL_AND_MICROSATELLITE_INSTABILITY_IN_COLORECTAL_CANCER	17	0.023 , 0.015	±
WP_MIRNA_REGULATION_OF_DNA_DAMAGE_RESPONSE ⋆	15	0.03252	+
WP_APOPTOSIS_MODULATION_AND_SIGNALING ⋆⋆	14	0.016 , 0.015	±
WP_NONALCOHOLIC_FATTY_LIVER_DISEASE	14	0.021	−
WP_APOPTOSIS ⋆⋆	13	0 , 0	±
WP_ATM_SIGNALING_PATHWAY ⋆	13	0.01984	+
HALLMARK_APOPTOSIS ⋆⋆	13	0.021 , 0.019	±
WP_TNFALPHA_SIGNALING_PATHWAY ⋆	12	0.03279 , 0.018	±
WP_ACUTE_VIRAL_MYOCARDITIS	11	0.014 , 0.010	±
WP_HOSTPATHOGEN_INTERACTION_OF_HUMAN_CORONAVIRUSES_APOPTOSIS ⋆⋆	8	0,0	±
WP_REGULATION_OF_APOPTOSIS_BY_PARATHYROID_HORMONERELATED_PROTEIN ⋆⋆	8	0.002 , 0	±
PID_BETA_CATENIN_NUC_PATHWAY	8	0 , 0	±
WP_TP53_NETWORK ⋆	6	0.006 , 0	±
WP_NANOMATERIAL_INDUCED_APOPTOSIS ⋆⋆	5	0 , 0	±
WP_LNCRNA_IN_CANONICAL_WNT_SIGNALING_AND_COLORECTAL_CANCER	5	0.016	−
WP_NCRNAS_INVOLVED_IN_WNT_SIGNALING_IN_HEPATOCELLULAR_CARCINOMA	5	0.006, 0.019	±
HALLMARK_IL2_STAT5_SIGNALING	5	0.004 , 0.002	±
PID_CASPASE_PATHWAY ⋆⋆	5	0.004 , 0	±
WP_COMPLEMENT_SYSTEM_IN_NEURONAL_DEVELOPMENT_AND_PLASTICITY	4	0.002,0.002	±
WP_WNTBETACATENIN_SIGNALING_PATHWAY_IN_LEUKEMIA	4	0.028 , 0.016	±
WP_LIPID_METABOLISM_PATHWAY	3	0 , 0	±
WP_LEPTIN_AND_ADIPONECTIN	2	0.008 , 0.004	±

Supplementary Table S7: Gene sets found to be enriched in the $\boldsymbol{\beta}_{\text{GraphR}}$ network estimations made by GraphR to a significance of nominal p-value < 0.05 and FDR q-val < 0.25. Polarity indicates whether the gene set was found enriched in the positive correlative structure (+), the negative (−) or in both (±). Where gene sets are found enriched in both correlative structures, the nominal p-values are given as ‘ + , − ’ . ⋆ indicates a relation to the epidermal growth factor receptor (EGFR) gene family and ⋆⋆ indicates a relation to ErbB4, which is part of the EGFR protein family.

**Gene Set**	**size**	**nom. p-val**	**polarity**
WP_EGFEGFR_SIGNALING_PATHWAY ⋆	25	0.012	+
WP_ERBB_SIGNALING_PATHWAY ⋆⋆	23	0 , 0.014	±
PID_ERBB1_DOWNSTREAM_PATHWAY ⋆⋆	18	0.006	−
WP_ENDOMETRIAL_CANCER	16	0.004	+
WP_ANDROGEN_RECEPTOR_SIGNALING_PATHWAY	15	0.013	+
WP_PHYSICOCHEMICAL_FEATURES_AND_TOXICITYASSOCIATED_PATHWAYS	15	0.018	+
WP_WNT_SIGNALING_PATHWAY	12	0.011	+
WP_FOLLICLE_STIMULATING_HORMONE_FSH_SIGNALING_PATHWAY	11	0.004	+
WP_ALPHA_6_BETA_4_SIGNALING_PATHWAY	10	0.001, 0.003	±
WP_SYNAPTIC_SIGNALING_PATHWAYS_ASSOCIATED_WITH_AUTISM_SPECTRUM_DISORDER	9	0.008	+
WP_EXTRACELLULAR_VESICLEMEDIATED_SIGNALING_IN_RECIPIENT_CELLS	9	0.007, 0.003	±
WP_REGUCALCIN_IN_PROXIMAL_TUBULE_EPITHELIAL_KIDNEY_CELLS	8	0.001	+
WP_TARGET_OF_RAPAMYCIN_TOR_SIGNALING	8	0.007	+
WP_EBOLA_VIRUS_INFECTION_IN_HOST	7	0.017	+
WP_TGFBETA_SIGNALING_IN_THYROID_CELLS_FOR_EPITHELIALMESENCHYMAL_TRANSITION	7	0.026	+
PID_AJDISS_2PATHWAY	6	0	−
WP_HEART_DEVELOPMENT	6	0.001	+
WP_RELATIONSHIP_BETWEEN_INFLAMMATION_COX2_AND_EGFR⋆	6	0.015	+
WP_STEROL_REGULATORY_ELEMENTBINDING_PROTEINS_SREBP_SIGNALING	6	0	+
PID_VEGFR1_PATHWAY ⋆	5	0	+
PID_ALK1_PATHWAY	4	0.003	+
WP_ECTODERM_DIFFERENTIATION	4	0.008	+

References

Claudio Busatto and Francesco Claudio Stingo. Inference of multiple high-dimensional networks with the graphical horseshoe prior. *Journal of Computational and Graphical Statistics*, 34(4):1202–1213, 2025. doi: 10.1080/10618600.2024.2435480.

Liying Chen, Satwik Acharyya, Chunyu Luo, Yang Ni, and Veerabhadran Baladandayuthapani. A probabilistic modeling framework for genomic networks incorporating sample heterogeneity. *Cell Reports Methods*, 5(2), 2025.

Zehang Richard Li and Tyler H McCormick. An expectation conditional maximization approach for gaussian graphical models. *Journal of Computational and Graphical Statistics*, 28(4):767–777, 2019.

Yang Ni, Francesco C Stingo, and Veerabhadran Baladandayuthapani. Bayesian covariate-dependent gaussian graphical models with varying structure. *Journal of Machine Learning Research*, 23(242): 1–29, 2022.

Yabo Niu, Yang Ni, Debdeep Pati, and Bani K Mallick. Covariate-assisted bayesian graph learning for heterogeneous data. *Journal of the American Statistical Association*, 119(547):1985–1999, 2024.

Damian Szklarczyk, Rebecca Kirsch, Mikaela Koutrouli, Katerina Nastou, Farrokh Mehryary, Radja Hachilif, Annika L Gable, Tao Fang, Nadezhda T Doncheva, Sampo Pyysalo, et al. The string database in 2023: protein–protein association networks and functional enrichment analyses for any sequenced genome of interest. *Nucleic acids research*, 51(D1):D638–D646, 2023.

University of Cambridge Research Computing Services. High performance computing. `https://www.hpc.cam.ac.uk/index.php/high-performance-computing`, 2025. Accessed: 2026-01-05.

Hao Wang. Bayesian graphical Lasso models and efficient posterior computation. *Bayesian Analysis*, 7 (4):867–886, 2012.

Hao Wang. Scaling it up: Stochastic search structure learning in graphical models. *Bayesian Analysis*, 10(2):351–377, 2015.

Xiaoyue Xi and Hélène Ruffieux. A modeling framework for detecting and leveraging node-level information in Bayesian network inference. *Biostatistics*, 26(1):kxae021, 06 2024. ISSN 1465-4644. doi: 10.1093/biostatistics/kxae021. URL `https://doi.org/10.1093/biostatistics/kxae021`.
